# The Golgi apparatus acts as a platform for TBK1 activation after viral RNA sensing

**DOI:** 10.1186/s12915-016-0292-z

**Published:** 2016-08-18

**Authors:** Marie Pourcelot, Naima Zemirli, Leandro Silva Da Costa, Roxane Loyant, Dominique Garcin, Damien Vitour, Ivana Munitic, Aimé Vazquez, Damien Arnoult

**Affiliations:** 1 INSERM, UMR_S 1197, Hôpital Paul Brousse, Villejuif, France; 2 Université Paris-Saclay, Paris, France; 3 Equipe Labellisée Ligue contre le Cancer, Villejuif, France; 4 Department of Microbiology and Molecular Medicine, Faculty of Medicine, University of Geneva, Geneva, Switzerland; 5 ANSES, INRA, ENVA, UPEC, UMR_1161 Virology, LabEx IBEID, Maisons-Alfort, France; 6 Laboratory of Molecular Immunology, Department of Biotechnology, University of Rijeka, Rijeka, Croatia

## Abstract

**Background:**

After viral infection and the stimulation of some pattern-recognition receptors, TANK-binding kinase I (TBK1) is activated by K63-linked polyubiquitination followed by *trans*-autophosphorylation. While the activated TBK1 induces type I interferon production by phosphorylating the transcription factor IRF3, the precise molecular mechanisms underlying TBK1 activation remain unclear.

**Results:**

We report here the localization of the ubiquitinated and phosphorylated active form of TBK1 to the Golgi apparatus after the stimulation of RIG-I-like receptors (RLRs) or Toll-like receptor-3 (TLR3), due to TBK1 K63-linked ubiquitination on lysine residues 30 and 401. The ubiquitin-binding protein optineurin (OPTN) recruits ubiquitinated TBK1 to the Golgi apparatus, leading to the formation of complexes in which TBK1 is activated by *trans*-autophosphorylation. Indeed, OPTN deficiency in various cell lines and primary cells impairs TBK1 targeting to the Golgi apparatus and its activation following RLR or TLR3 stimulation. Interestingly, the Bluetongue virus NS3 protein binds OPTN at the Golgi apparatus, neutralizing its activity and thereby decreasing TBK1 activation and downstream signaling.

**Conclusions:**

Our results highlight an unexpected role of the Golgi apparatus in innate immunity as a key subcellular gateway for TBK1 activation after RNA virus infection.

**Electronic supplementary material:**

The online version of this article (doi:10.1186/s12915-016-0292-z) contains supplementary material, which is available to authorized users.

## Background

The innate immune response is the first line of defense against microbial pathogens, including viruses. After infection, the replication of a virus within host cells generates molecular signatures known as pathogen-associated molecular patterns. The antiviral immune response is dependent on germline-encoded pattern-recognition receptors (PRRs), which sense the presence of viral nucleic acids and trigger a series of signaling pathways leading to the rapid production of pro-inflammatory cytokines and type I interferons (IFNα/IFNβ) [1, 2]. Once secreted, type I IFNs stimulate the transcription of IFN-stimulated genes to prevent the virus from spreading and to activate the adaptive immune response [3]. The loss of type I IFN signaling leads to severe immunodeficiency with a high susceptibility to viral infection [4].

The various classes of PRRs involved in virus detection include endosomal Toll-like receptors (TLRs), cytosolic DexD/H-box retinoic acid-inducible gene-I (RIG-I)-like receptors (RLRs) and cytosolic DNA receptors [1, 2]. All antiviral PRRs induce type I IFN expression, but the signaling components involved differ between PRRs. However, one common feature of all these signaling pathways is the recruitment of adaptor proteins to form a scaffold with the cellular ubiquitin ligases TRAFs. For instance, viral dsRNA in endosomes is detected by TLR3, whereas viral RNA in the cytosol is detected by RLRs, leading to the recruitment of TRIF and MAVS (this protein being anchored in the mitochondrial outer membrane), respectively. With the assistance of TRAFs and through polyubiquitination, these two adaptors then activate two cytosolic protein kinase complexes, one consisting of the ‘non-canonical’ inhibitor of nuclear factor κB kinase (IKK)-related TANK binding kinase 1 (TBK1) or its close homolog IKKε, associated with various adaptor proteins, and the other containing IKKα, IKKβ, and NEMO [1, 5, 6]. The IKK complex activates NF-κB, promoting the production of pro-inflammatory cytokines, and NF-κB activation requires the degradation of cytoplasmic inhibitors. By contrast, the transcription factors IRF3 and IRF7 are directly activated in the cytoplasm through TBK1-mediated phosphorylation, leading to their dimerization, translocation to the nucleus and the initiation of type I IFN production [3, 7].

TBK1 is a key regulator of type I IFN production. Indeed, the loss of TBK1 has a profound impact on type I IFN induction after viral infection [7–9]. TBK1 is constitutively expressed and TBK1 deficiency is embryo-lethal, due to the occurrence of high levels of hepatic apoptosis, a phenotype very similar to that of IKKβ-deficient mice [10]. TBK1 activity is regulated by phosphorylation of the serine 172 residue within the classical kinase activation loop. The mechanisms of TBK1 activation are not clearly understood. Genetic and pharmacological inhibition studies have indicated that TBK1 can be activated by IKKβ but that activation by *trans*-autophosphorylation is more likely [11, 12]. Post-translational modifications of some lysine residues of TBK1 through the addition of K63-linked polyubiquitin chains have been shown to be required for TBK1 activation and type I IFN production after viral infections [13, 14]. Moreover, it has been suggested that TBK1 autoactivation is dependent on the subcellular location of TBK1, with various adaptor proteins each directing TBK1 to discrete signaling complexes for different cellular responses [12, 15, 16].

We report here the targeting of ubiquitinated TBK1 to the Golgi apparatus after RLR or TLR3 stimulation, through interaction with optineurin (OPTN), an ubiquitin-binding adaptor protein [17]. OPTN senses the K63-linked polyubiquitin chains on TBK1, triggering the formation of TBK1-OPTN complexes, in which TBK1 is activated by *trans*-autophosphorylation. TBK1 then phosphorylates IRF3 to promote type I IFN production to establish an antiviral response. Interestingly, the Bluetongue virus NS3 protein neutralizes OPTN activity at the Golgi apparatus, thereby decreasing TBK1 activation and downstream signaling. Our observations thus reveal an unexpected new function of the Golgi apparatus in innate immunity.

## Results

### The active form of TBK1 localizes to the Golgi apparatus after RLR or TLR3 activation

**Fig. 1:**
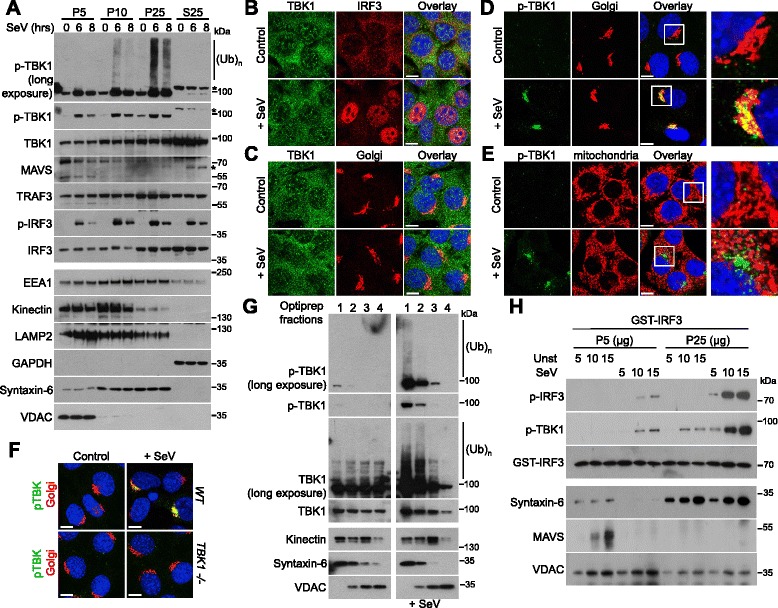
Localization of the active form of TBK1 at the Golgi apparatus after RLR stimulation. **a** MEFs were either left unstimulated or infected with Sendai virus (SeV) for 6 or 8 h. MEFs were then fractionated as described in Additional file 1A, and samples were analyzed by immunoblotting with antibodies against the indicated proteins. EEA1, kinectin, LAMP2, GAPDH, syntaxin-6, and VDAC served as loading and purity controls for endosomes, the endoplasmic reticulum, lysosomes, the cytosol, the Golgi apparatus, and mitochondria, respectively. (Ub)n, polyubiquitin. * Indicates non-specific bands. **b**–**e** MEFs were either left unstimulated (control) or infected with SeV for 6 h (+ SeV). The indicated proteins were then analyzed by immunofluorescence. The Golgi apparatus was stained with an antibody raised against GM130, whereas the mitochondria were identified by labeling with an antibody against cytochrome c. Scale bars, 10 μm. On the right, enlargement of the framed zone in the overlay. **f** WT or TBK1^–/–^ MEFs were either left unstimulated (control) or infected with SeV for 6 h (+ SeV). p-TBK1^S172^ staining was then analyzed by immunofluorescence. The Golgi apparatus was stained with an antibody raised against GM130. Scale bars, 10 μm. **g** Crude heavy membrane fractions from uninfected or SeV-infected MEFs were fractionated on OptiPrep density gradients (fractions range from 1 at the top to 4 at the bottom) and analyzed by immunoblotting with antibodies against the indicated proteins. (Ub)n, polyubiquitin. **h** Increased concentrations of mitochondria (P5) or Golgi (P25)-enriched fractions of unstimulated (Unst) or SeV-infected HEK293T cells were incubated with recombinant GST-IRF3 in the presence of ATP. The degree of IRF3 phosphorylation was determined by immunoblotting

We recently reported that ubiquitinated transmitters involved in NF-κB activation accumulate at the surface of the endoplasmic reticulum [18]. This raised questions as to whether some endomembranes might also act as sites of accumulation for ubiquitinated transmitters involved in activation of the transcription factor IRF3. To explore this hypothesis, murine embryonic fibroblasts (MEFs) were infected with Sendai virus to stimulate the RLR signaling pathway. Differential centrifugation was then used to separate the different membrane fractions, as indicated in Additional file 1A. With this approach, while a significant proportion of TBK1 was found in the cytosolic fraction (S25), unexpectedly, the ubiquitinated and phosphorylated active form of TBK1 (p-TBK1^S172^) was detected mostly in the Golgi-enriched fraction (P25), and this TBK1 activation was associated with the phosphorylation of its substrate IRF3 (Fig. 1a). Immunofluorescence confirmed that IRF3 was activated, as it was translocated to the nucleus after infection (Fig. 1b). TBK1 staining was not affected by RLR activation (Fig. 1c), whereas p-TBK1^S172^ was detected at the Golgi apparatus (Fig. 1d and Additional file 1B), but not at mitochondria (Fig. 1e). Similarly, the infection of HeLa cells with Sendai virus led to a detection of the active form of TBK1 at the Golgi apparatus (Additional file 2A–E). TBK1^–/–^ MEFs confirmed the specificity of the used antibody raised against p-TBK1^S172^ (Fig. 1f) and, at later time points post-infection, we observed that the active form of TBK1 accumulates very close to the Golgi apparatus, at the centrosome (Additional file 1D and E). Importantly, RLR activation promoted the K63-linked polyubiquitination of TBK1, as previously reported (Additional file 2F) [13].

For further confirmation of the presence of the active form of TBK1 at the Golgi apparatus after RLR stimulation, cell fractions from control or infected cells were separated on Optiprep gradients to yield fractions enriched in Golgi apparatus, endoplasmic reticulum, or mitochondria. Unmodified TBK1 was detected in multiple fractions, whereas the ubiquitinated and phosphorylated protein was essentially limited to fractions enriched in the Golgi protein syntaxin-6 (Fig. 1g). p-TBK1^S172^ was also detected, using discontinuous density gradients, in Golgi membranes isolated from Sendai virus-infected cells (Additional file 1C).

Finally, to demonstrate that the active form of TBK1 was mostly located at the Golgi apparatus but not at the mitochondria after RLR stimulation, increased concentrations of mitochondria- (P5) or Golgi-enriched (P25) fractions of control or Sendai virus-infected cells were incubated with recombinant IRF3 in the presence of ATP. The mitochondria-enriched fraction of infected cells triggered the phosphorylation of recombinant IRF3, but this phosphorylation was significantly weaker than that observed with the Golgi-enriched fraction, in which most of the p-TBK1^S172^ was found (Fig. 1h). Our results are consistent with the reports that TBK1 does not sediment in the high molecular weight fractions together with the prion-like aggregates of MAVS at the mitochondria in response to Sendai virus infection [19, 20].

**Fig. 2:**
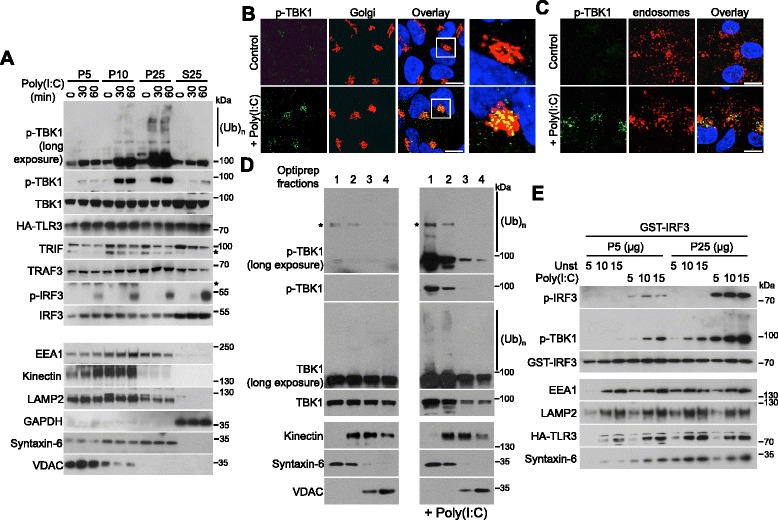
The active form of TBK1 localizes at the Golgi apparatus after TLR3 stimulation. **a** HEK293T cells stably expressing HA-TLR3 were either left untreated or stimulated with poly(I:C) (10 μg/mL) for 30 or 60 minutes. Cells were then fractionated as described in Additional file 1A, and samples were analyzed by immunoblotting with antibodies against the indicated proteins. EEA1, kinectin, LAMP2, GAPDH, syntaxin-6, and VDAC served as loading and purity controls for endosomes, the endoplasmic reticulum, lysosomes, the cytosol, the Golgi apparatus, and mitochondria, respectively. (Ub)n, polyubiquitin. * Indicates non-specific bands. **b**, **c** HEK293T cells stably expressing HA-TLR3 were either left untreated (control) or stimulated with poly(I:C) (10 μg/mL) for 1 h (+ Poly(I:C)). The indicated proteins were then analyzed by immunofluorescence. The Golgi apparatus was stained with an antibody against GM130, and an antibody against EEA1 was used to stain endosomes. Scale bars, 10 μm. In b, on the right, enlargement of the framed zone in the overlay. **d** Crude heavy membrane fractions from unstimulated or poly(I:C)-treated HEK293T cells stably expressing HA-TLR3 were fractionated on OptiPrep density gradients (fractions range from 1 at the top to 4 at the bottom) and analyzed by immunoblotting with antibodies against the indicated proteins. (Ub)n, polyubiquitin. * Indicates non-specific bands. **e** Increased concentrations of mitochondria- (P5) or Golgi (P25)-enriched fractions of unstimulated (Unst) or poly(I:C)-treated HEK293T cells stably expressing HA-TLR3 were incubated with recombinant GST-IRF3 in the presence of ATP. The degree of IRF3 phosphorylation was determined by immunoblotting

Within the endosomes, viral dsRNA is detected by TLR3, and the activation of this PRR leads to TBK1 activation after recruitment of the TRIF adaptor [2]. We therefore investigated whether active TBK1 was also found at the Golgi apparatus after TLR3 stimulation. HEK293 cells stably expressing HA-tagged TLR3 were stimulated with poly(I:C), an analog of viral dsRNA, and then differential centrifugation was performed to separate the various membrane fractions, as in Additional file 1A. As observed after RLR stimulation, the ubiquitinated and phosphorylated active form of TBK1 was mostly present in the Golgi-enriched fraction (Fig. 2a). Immunofluorescence studies confirmed the presence of active TBK1 at the Golgi apparatus but not at the endosomes after TLR3 stimulation (Fig. 2b, c). Optiprep gradients further demonstrated that p-TBK1^S172^ was concentrated in the Golgi-enriched fraction (Fig. 2d) and that this fraction could phosphorylate recombinant IRF3 in vitro (Fig. 2e). Thus, regardless of the viral pathogen-associated molecular pattern sensing location, i.e. cytoplasmic RLR or endosomal TLR, the active form of TBK1 is localized at the Golgi apparatus.

### Potent RLR activation triggers TBK1 targeting and aggregation at the Golgi apparatus

**Fig. 3:**
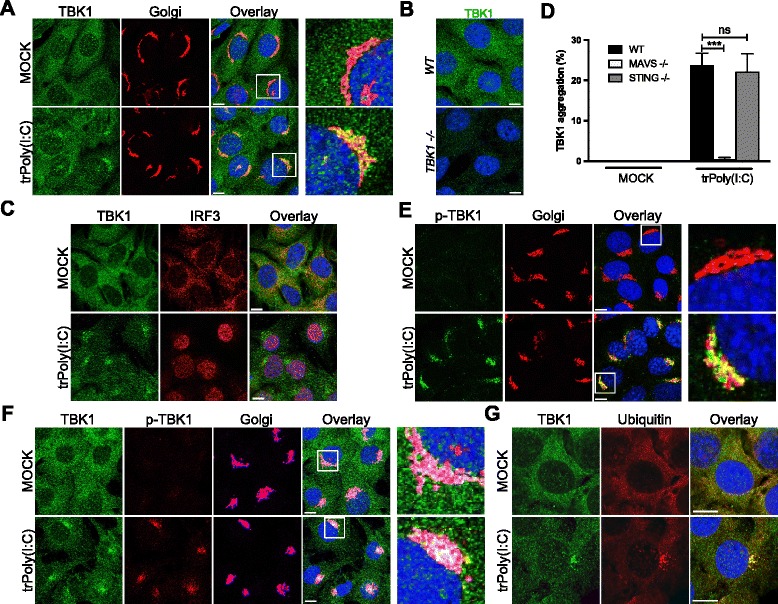
TBK1 forms aggregates at Golgi apparatus following potent RLR activation. **a** MEFs were either left untreated (MOCK) or transfected with HMW poly(I:C) (5 μg/mL) for 2 h (trPoly(I:C)). TBK1 staining was then analyzed by immunofluorescence. The Golgi apparatus was identified by labeling with an antibody raised against GM130. Scale bars, 10 μm. On the right, enlargement of the framed zone in the overlay. **b** WT or TBK1^–/–^ MEFs were stained with an antibody raised against TBK1, then analyzed by immunofluorescence analysis. **c** MEFs were either left untreated (MOCK) or transfected with HMW poly(I:C) (5 μg/mL) for 2 h (trPoly(I:C)). The indicated proteins were analyzed by immunofluorescence analysis with specific antibodies. Scale bars, 10 μm. **d** WT, MAVS^–/–^, or STING^–/–^ MEFs were either left untreated (MOCK) or transfected with HMW poly(I:C) (5 μg/mL) for 4 h (trPoly(I:C)). TBK1 aggregation was assessed by immunofluorescence staining and aggregate counting. The data shown are means ± SD from three independent experiments (300 cells were counted per condition). ***P < 0.001 versus WT MEFs (Student’s *t* test). ns, not significant. **e**–**g** MEFs were either left untreated (MOCK) or transfected with HMW poly(I:C) (5 μg/mL) for 2 or 4 h (trPoly(I:C)). The indicated proteins were analyzed by immunofluorescence analysis with specific antibodies. The Golgi apparatus was stained with an antibody against GM130. Scale bars, 10 μm

After RLR stimulation, TBK1 activation is initiated at the mitochondria through the essential adaptor MAVS [2, 6]. However, active TBK1 was detected at the Golgi apparatus following Sendai virus infection suggesting the targeting of TBK1 to this organelle. This redistribution of TBK1 was further studied by stimulating RLRs in MEFs by transfection with poly(I:C), which is sensed in the cytosol by RIG-I or melanoma differentiation-associated gene-5, in a size-dependent manner [21]. Interestingly, transfection with low- or high-molecular weight poly(I:C) for 2 hours triggered a TBK1 aggregation at the Golgi apparatus (Fig. 3a). TBK1 staining was lost in TBK1^–/–^ MEFs (Fig. 3b) confirming the specificity of the anti-TBK1 antibody and this TBK1 aggregation was also detected with another antibody raised against the kinase (Additional file 3A). TBK1 aggregation was associated with the nuclear translocation of IRF3 (Fig. 3c) and these aggregates do not co-localize with the autophagosomes (data not shown). Interestingly, 4 hours after poly(I:C) transfection, TBK1 accumulated at the centrosome, which is surrounded by the Golgi apparatus next to the nucleus (Additional file 3B, C). TBK1 aggregation was completely inhibited by transfection with poly(I:C) in MAVS^–/–^ MEFs but not in STING^–/–^ MEFs (Fig. 3d), STING being a crucial adaptor involved in cytosolic DNA sensing pathways anchored in the endoplasmic reticulum [22].

After the transfection of cells with poly(I:C), p-TBK1^S172^ was first detected at the Golgi apparatus (Fig. 3e) next at the centrosome (Additional file 3D and E) as observed during Sendai virus infection. Finally, as expected, the TBK1 aggregates consisted of the phosphorylated and ubiquitinated active form of the kinase (Fig. 3f, g and Additional file 4).

### Ubiquitination targets TBK1 to the Golgi apparatus for activation

**Fig. 4:**
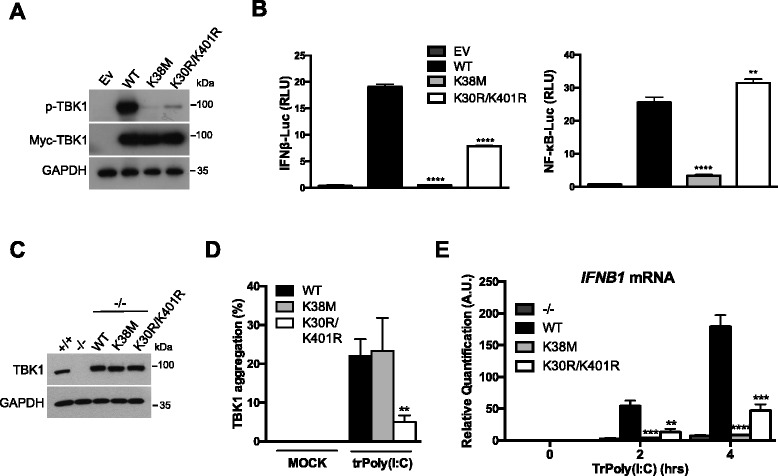
Ubiquitination promotes TBK1 targeting to the Golgi apparatus for activation. **a** HEK293T cells were transfected with an empty vector (Ev) or with plasmids encoding myc-tagged WT TBK1 (WT), TBK1^K38M^ (K38M), or TBK1^K30R/K401R^ (K30R/K401R). After 16 h, TBK1 activation and exogenous TBK1 expression were assessed by immunoblotting with anti-p-TBK1^S172^ and anti-myc antibodies, respectively. GAPDH was used as a loading control. **b** HEK293T cells were transfected with either an IFNβ promoter reporter or an NF-κB reporter, together with the *Renilla* luciferase gene as an internal control. In parallel, the cells were also transfected with an Ev or with plasmids encoding myc-tagged WT TBK1 (WT), TBK1^K38M^ (K38M), or TBK1^K30R/K401R^ (K30R/K401R). Luciferase assays were performed 24 h after transfection and the results were normalized against *Renilla* luciferase activity. The data shown are means ± SD from three independent experiments (analysis of variance and comparison with WT TBK1 in Student’s *t* test). RLU, relative luminescence units. **c** Immunoblotting analysis of TBK1^–/–^ MEFs reconstituted with WT TBK1, TBK1^K38M^ (K38M), or TBK1^K30R/K401R^ (K30R/K401R). As controls, TBK1^+/+^ and TBK1^–/–^ MEFs are shown. **d** TBK1^–/–^ MEFs reconstituted with WT TBK1 or mutants were either left untreated (MOCK) or transfected with HMW poly(I:C) (5 μg/mL) for 4 h (trPoly(I:C)). TBK1 aggregation was then assessed by immunofluorescence staining and counting of the aggregates. The data shown are means ± SD from three independent experiments (300 cells were counted per condition). **0.001 < *P* < 0.01 versus MEFs reconstituted with WT TBK1 (Student’s *t* test). **e** The reconstituted MEFs described in (c) and the initial TBK1^–/–^ MEFs were transfected with HMW poly(I:C) (5 μg/mL) for 0, 2, and 4 h (trPoly(I:C)). *IFNB1* mRNA levels were then assessed by RT-qPCR with normalization against GAPDH. The data shown are means ± SD from three independent experiments (analysis of variance and comparison with WT TBK1-reconstituted MEFs in Student’s *t* test). AU, arbitrary unit

The K63-linked polyubiquitination of TBK1 on lysines 30 and 401 is required for the activation of this kinase [14]. Thus, whereas the overexpression of wildtype (WT) TBK1 led to activation of the kinase through *trans*-autophosphorylation, TBK1 activation was impaired after overexpression of the TBK1^K30R/K401R^ double mutant (Fig. 4a). The expression of a kinase-inactive mutant (K38M mutant) as a positive control did not trigger TBK1 activation (Fig. 4a). Consistent with these findings, significantly weaker stimulation of the IFNβ promoter was observed in luciferase assays after the overexpression of TBK1^K30R/K401R^ than with WT TBK1, and the promoter was not activated with TBK1^K38M^ (Fig. 4b). Interestingly, NF-κB activation was not weaker with the polyubiquitination mutant than for the WT, whereas this activation was prevented with the kinase-inactive mutant (Fig. 4b).

Following our detection of ubiquitinated active TBK1 at the Golgi apparatus, we hypothesized that the targeting of TBK1 to the Golgi apparatus might be impaired in the absence of ubiquitination. We tested this hypothesis by reconstituting TBK1^–/–^ MEFs with WT or mutant TBK1 constructs (Fig. 4c), and then investigating TBK1 aggregation after transfection with poly(I:C). Stimulation triggered TBK1 aggregation in cells reconstituted with WT TBK1, but this aggregation was significantly impaired with the K30R/K401R polyubiquitination mutant (Fig. 4d). In parallel, the expression of the IRF3 target gene *IFNB1* was analyzed. Transfection with poly(I:C) increased *IFNB1* mRNA levels in cells reconstituted with WT TBK1, but this response was abolished with the polyubiquitination mutant (Fig. 4e), as previously described [14]. We also reconstituted TBK1^–/–^ MEFs with the kinase-inactive mutant. TBK1 aggregation was unaffected (Fig. 4d), but the transcriptional response was completely inhibited (Fig. 4e). Thus, TBK1 polyubiquitination on conserved lysines 30 and 401 targets TBK1 to the Golgi apparatus in a process linked to the phosphorylation of the Ser172 residue in the kinase activation loop after dimerization [14]. These observations are consistent with the hypothesis developed from findings for structural studies concerning the key role of cellular localization in the activation of TBK1 [12].

### OPTN is required for optimal TBK1 activation after RLR or TLR3 stimulation

**Fig. 5:**
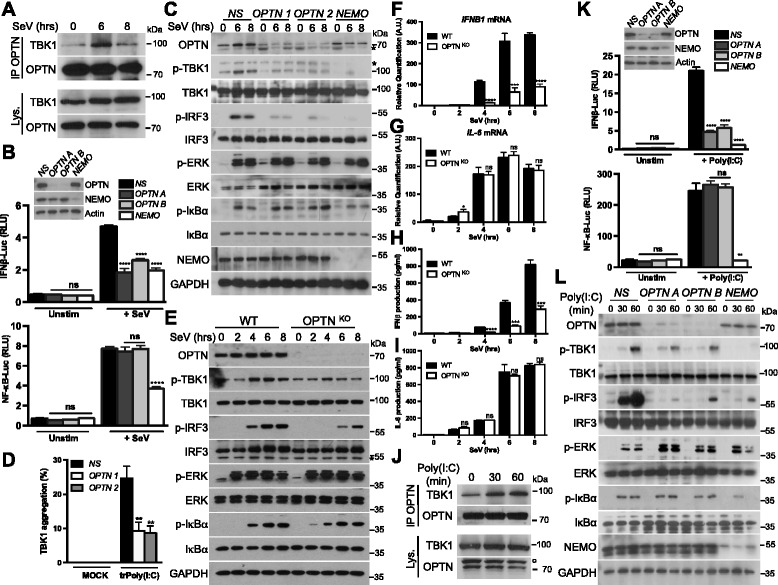
OPTN silencing impairs TBK1 activation after RLR or TLR3 activation. **a** HEK293T cells were either left unstimulated or infected with Sendai virus (SeV) for 6 and 8 h. Cell lysates (Lys.) were subjected to immunoprecipitation (IP) with an antibody against OPTN. Samples were then analyzed by immunoblotting with antibodies against the indicated proteins. **b** HEK293T cells were transfected with a control non-specific siRNA (NS) or with two individual OPTN-specific siRNAs (OPTN A and OPTN B) or a NEMO-specific siRNA. The cells were then transfected, 48 h later, with an IFNβ promoter reporter or with a NF-κB reporter and the *Renilla* luciferase gene as an internal control. Then, 24 h after transfection, cells were either left unstimulated (Unstim) or infected with Sendai virus (+ SeV) for 7 h. Luciferase assays were performed and the results were normalized against *Renilla* luciferase activity. The data shown are means ± SD from three independent experiments. ****P < 0.0001 versus the NS siRNA-transfected cells (Student’s *t* test). RLU, relative luminescence units. ns, not significant. **c** MEFs were transfected with a control non-specific siRNA (NS) or with two individual OPTN-specific siRNAs (OPTN 1 and OPTN 2) or a NEMO-specific siRNA. Then, 72 h later, cells were either left unstimulated or infected with SeV for 6 or 8 h. Cell lysates were analyzed by immunoblotting with antibodies against the indicated proteins. * Indicates non-specific bands. **d** MEFs were transfected with a control non-specific siRNA (NS) or with two individual OPTN-specific siRNAs (OPTN 1 and OPTN 2). Then, 72 h later, cells were either left untreated (MOCK) or transfected with high molecular weight poly(I:C) (5 μg/mL) for 4 h (trPoly(I:C)). TBK1 aggregation was assessed by immunofluorescence staining and counting of the aggregates. The data shown are means ± SD from three independent experiments (300 cells were counted per condition). **0.001 < *P* < 0.01 versus the NS siRNA-transfected cells (Student’s *t* test). **e** WT or OPTN KO HeLa cells were infected with SeV for the indicated times. Cell lysates were then analyzed by immunoblotting with antibodies against the indicated proteins. * Indicates a non-specific band. **f**–**i** WT or OPTN KO HeLa cells were infected with SeV for the indicated times. *IFNB1* and *IL-6* mRNA levels were then assessed by RT-qPCR with normalization against GAPDH (f, g), or the production of IFNβ and IL-6 was analyzed by ELISA in the cell supernatant (h, i). The data shown are means ± SD from three independent experiments (analysis of variance and comparison with WT HeLa cells in Student’s *t* test). ns, not significant; AU, arbitrary unit. **j** HEK293T cells stably expressing TLR3 (HEK293-TLR3) were either left unstimulated or stimulated with poly(I:C) (10 μg/mL) for 30 or 60 minutes. Cell lysates (Lys.) were subjected to immunoprecipitation (IP) with an antibody against OPTN. Samples were then analyzed by immunoblotting with antibodies against the indicated proteins. ° Corresponds to TBK1 detection from a previous immunoblot. **k** HEK293-TLR3 cells were transfected with a control non-specific siRNA (NS) or with two individual OPTN-specific siRNAs (OPTN A and OPTN B) or a NEMO-specific siRNA. The cells were then transfected, 48 h later, with an IFNβ promoter reporter or with a NF-κB reporter and the *Renilla* luciferase gene as an internal control. Then, 24 h after transfection, cells were either left unstimulated (Unstim) or stimulated with poly(I:C) (10 μg/mL) for 7 h. Luciferase assays were performed and the results were normalized against *Renilla* luciferase activity. The data shown are means ± SD from three independent experiments (analysis of variance and comparison with the NS siRNA-transfected cells in Student’s *t* test). ns, not significant. **l** HEK293-TLR3 cells were transfected as in (k). Then, 72 h later, cells were either left unstimulated or stimulated with poly(I:C) (10 μg/mL) for 30 or 60 minutes. Cell lysates were then analyzed by immunoblotting with antibodies against the indicated proteins

A structural study has also suggested that the binding of polyubiquitin chains triggers the higher-order oligomerization of TBK1-adaptor complexes, resulting in the *trans*-autophosphorylation and activation of the kinase [11]. As observed after RLR or TLR3 stimulation, the ubiquitinated and phosphorylated active form of TBK1 was detected at the Golgi apparatus. This suggested that an adaptor of ubiquitinated TBK1 was present at this organelle to promote the activation of TBK1. We identified optineurin (OPTN) as a likely candidate for this role. Indeed, this protein has an ubiquitin-binding domain (UBD) and a C-terminal ubiquitin-binding zinc finger (ZF), and is located at the Golgi apparatus [17, 23]. It has also been reported to be required for optimal TBK1 activation [24–28]. Immunofluorescence studies with three different antibodies against OPTN confirmed that a part of this protein is located at the Golgi apparatus (Additional file 5A), as previously reported [17, 23]. Moreover, OPTN was also detected in isolated Golgi membranes (Additional file 1C). Following infection with Sendai virus, leading to RLR activation, an increase in the association of TBK1 with OPTN was detected (Fig. 5a) and p-TBK1^S172^ was found in complex with OPTN in the Golgi-enriched fraction (Additional file 5B).

We then carried out assays on HEK293T cells with a luciferase reporter under the control of the IFNβ promoter or driven by three copies of an NF-κB enhancer. We found that stimulation of the IFNβ promoter after RLR activation is impaired in cells in which OPTN expression was knocked down with two different siRNAs, whereas NF-κB activation was unaffected (Fig. 5b). These results were confirmed with three other OPTN siRNAs (Additional file 6A). As a control, NEMO expression was knocked down, as this protein activates both the NF-κB and IRF signaling pathways downstream from MAVS [29] and its loss inhibited both pathways (Fig. 5b). Biochemical studies in both HEK293T and HeLa cells confirmed that transfection with siRNAs against OPTN impairs TBK1 activation and subsequent signaling after RLR stimulation, but has no effect on the NF-κB and ERK pathways (Additional files 6B and C). The infection of OPTN siRNA-transfected cells with another RNA virus, such as the vesicular stomatitis virus, to activate the RLRs, yielded similar results (Additional files 6D and E). Similarly, OPTN silencing in MEFs impaired TBK1 activation and IRF3 phosphorylation without altering NF-κB or ERK signaling after Sendai virus infection (Fig. 5c). Importantly, TBK1 aggregation at the Golgi apparatus after poly(I:C) transfection was prevented by the silencing of OPTN (Fig. 5d). This suggests that OPTN senses ubiquitinated TBK1 at the Golgi apparatus and promotes its activation, because the ubiquitination of TBK1 is required for its targeting to this organelle (Fig. 4).

OPTN expression was also abolished with CRISPR/Cas9 technology in HeLa cells and the cells were then infected with Sendai virus. As expected, the IRF3 signaling pathway was decreased in OPTN knockout cells, whereas the NF-κB and ERK pathways were not (Fig. 5e). Consequently, smaller amounts of IFNβ were produced and secreted, but no effect was observed on the NF-κB target IL-6 (Fig. 5f–i). Finally, the lack of OPTN did not affect the K63-linked polyubiquitination of TBK1 after RLR stimulation (Additional file 7).

As ubiquitinated active TBK1 was uncovered at the Golgi apparatus after TLR3 stimulation (Fig. 2), we also explored the possible involvement of OPTN in TBK1 activation downstream from this PRR. Hence, an increased association between OPTN and TBK1 was detected after TLR3 stimulation (Fig. 5j). We then silenced OPTN, and both luciferase assays and immunoblots showed that knocking down the levels of this protein selectively inhibited the IRF3 signaling pathway (Fig. 5k, l and Additional file 6F).

### OPTN insufficiency impairs TBK1 activation after RLR or TLR3 stimulation in primary cells

**Fig. 6:**
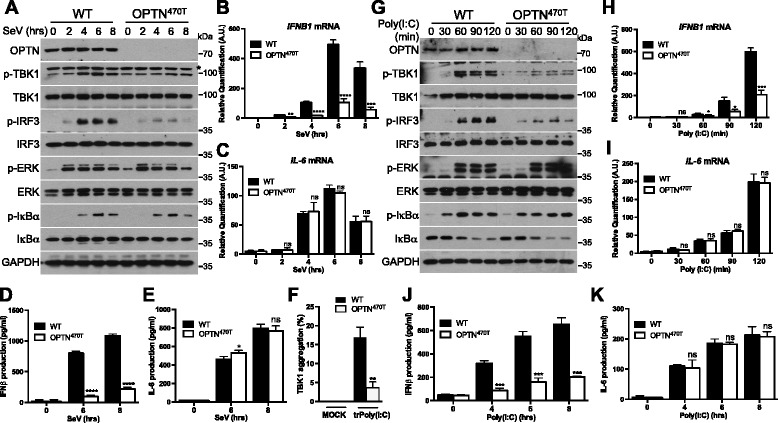
Impaired TBK1 activation after RLR or TLR3 stimulation in OPTN-deficient primary cells. **a** Primary MEFs isolated from WT or OPTN^470T^ mice were infected with Sendai virus (SeV) for the indicated times. Cell lysates were then analyzed by immunoblotting with antibodies against the indicated proteins. * Indicates a non-specific band. **b**, **c** Primary MEFs isolated from WT or OPTN^470T^ mice were infected with SeV for the indicated times. *IFNB1* and *IL-6* mRNA levels were assessed by RT-qPCR with normalization against GAPDH. The data shown are means ± SD from three independent experiments (analysis of variance and comparison with WT MEFs in Student’s *t* test). ns, not significant; AU, arbitrary unit. **d**, **e** Primary MEFs isolated from WT or OPTN^470T^ mice were either left unstimulated or infected with SeV for 6 or 8 h. The production of IFNβ and IL-6 was then analyzed by ELISA in the cell supernatant. The data shown are means ± SD from three independent experiments (analysis of variance and comparison with WT MEFs in Student’s *t* test). ns, not significant. **f** Primary MEFs isolated from WT or OPTN^470T^ mice were either left untreated (MOCK) or transfected with HMW poly(I:C) (5 μg/mL) for 4 h (trPoly(I:C)). TBK1 aggregation was assessed by immunofluorescence staining and aggregate counting. The data shown are means ± SD from three independent experiments (300 cells were counted per condition). **0.001 < *P* < 0.01 versus WT MEFs (Student’s *t* test). **g** BMDM isolated from WT or OPTN^470T^ mice were stimulated with poly(I:C) (1 μg/mL) for the indicated times. Cell lysates were then analyzed by immunoblotting with antibodies against the indicated proteins. **h**–**k** BMDM isolated from WT or OPTN^470T^ mice were stimulated with poly(I:C) (1 μg/mL) for the indicated times. *IFNB1* and *IL-6* mRNA levels were then assessed by RT-qPCR with normalization against GAPDH (h, i), or the production of IFNβ and IL-6 was analyzed by ELISA in the cell supernatant (j, k). The data shown are means ± SD from three independent experiments (analysis of variance and comparison with WT BMDM in Student’s *t* test). ns, not significant; AU, arbitrary unit

For further confirmation of the requirement of OPTN for TBK1 activation, we used primary cells from mice in which both the UBD and ZF domains of OPTN had been deleted (OPTN^470T^) [27]. The truncated protein was produced in smaller amounts than the WT protein, allowing to investigate the effects of both defective Ub binding and OPTN insufficiency [27]. First, primary MEFs were infected with Sendai virus. OPTN^470T^ MEFs displayed weaker TBK1 activation than WT MEFs, together with lower levels of phosphorylation of IRF3, with no effect on ERK or NF-κB signaling (Fig. 6a). Confirming these observations, levels of *IFNB1* mRNA and of IFNβ release were lower in the OPTN^470T^ MEFs, whereas the production and secretion of IL-6 were unaffected (Fig. 6b–e). Furthermore, consistent with OPTN being required for recruiting ubiquitinated TBK1 to the Golgi apparatus, significantly less TBK1 aggregation was observed with the mutated OPTN (Fig. 6f). Finally, the comparison of WT and OPTN^470T^ bone marrow derived macrophages (BMDM) stimulated with poly(I:C) also confirmed that OPTN positively regulates TBK1 activation and downstream signaling after TLR3 stimulation without influencing NF-κB or ERK signaling (Fig. 6g–k).

Together, our results suggest that OPTN recruits, at the Golgi apparatus, ubiquitinated TBK1 downstream from both RLRs and TLR3 in order to promote TBK1 activation and a signaling pathway resulting in the production of type I IFNs.

### The NS3 protein of the Bluetongue virus targets OPTN to dampen IRF3 signaling

Viruses have developed a battery of different strategies for overcoming the very sophisticated defense mechanisms of infected hosts. During the course of pathogen–host co-evolution, viruses have acquired an ability to inhibit the innate immune response by targeting host proteins [30]. Our results suggested that OPTN is important for TBK1 activation after RLR or TLR3 activation. We therefore hypothesized that there might be viral proteins capable of neutralizing the activity of OPTN, thereby preventing it from performing its function in innate immunity.

**Fig. 7:**
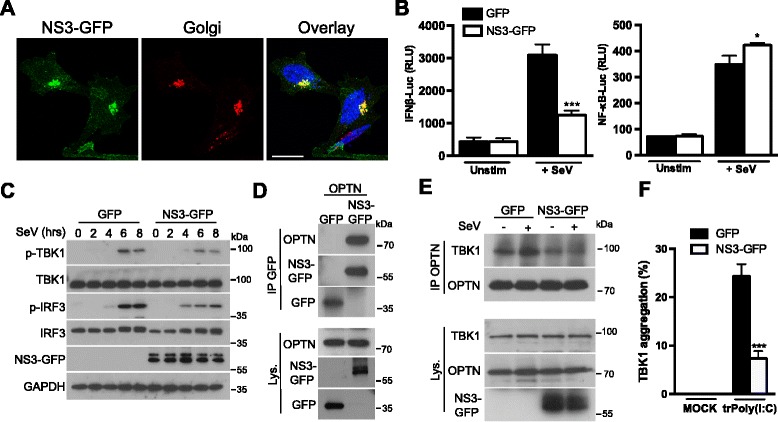
OPTN is targeted by the NS3 protein of the Bluetongue virus to dampen IRF3 signaling. **a** HeLa cells were transfected with a plasmid encoding NS3-GFP; 16 h later, the NS3-GFP localization was assessed by immunofluorescence analysis. The Golgi apparatus was stained with an antibody against GM130. Scale bars, 10 μm. **b** HEK293T cells were transfected with either an IFNβ promoter reporter or with a NF-κB reporter, together with the *Renilla* luciferase gene as an internal control. In parallel, the cells were also transfected with a plasmid encoding GFP or NS3-GFP. Then, 16 h after transfection, cells were either left unstimulated (Unstim) or infected with Sendai virus (+ SeV) for 7 h. Luciferase assays were then performed and the results were normalized against *Renilla* luciferase activity. The data shown are means ± SD from three independent experiments (analysis of variance and comparison with GFP-transfected cells in Student’s *t* test). RLU, relative luminescence units. **c** HEK293T cells were transfected with a plasmid encoding GFP or NS3-GFP. Then, 16 h after transfection, cells were infected with SeV for the indicated times. Cell lysates were analyzed by immunoblotting with antibodies against the indicated proteins. **d** HEK293T cells were transfected with a plasmid encoding OPTN and a plasmid encoding GFP or NS3-GFP. Then, 16 h after transfection, cell lysates (Lys.) were subjected to immunoprecipitation (IP) with an antibody against GFP. Samples were analyzed by immunoblotting with antibodies against the indicated proteins. **e** HEK293T cells were transfected with a plasmid encoding GFP or NS3-GFP. Then, 16 h after transfection, cells were either left unstimulated or infected with Sendai virus for 7 h. Cell lysates (Lys.) were then subjected to immunoprecipitation (IP) with an antibody against OPTN. Samples were analyzed by immunoblotting with antibodies against the indicated proteins. **f** MEFs were transfected with a plasmid encoding GFP or NS3-GFP. Then, 16 h after transfection, cells were either left untreated (MOCK) or transfected with HMW poly(I:C) (5 μg/mL) for 4 h (trPoly(I:C)). TBK1 aggregation was assessed by immunofluorescence staining and aggregate counting in GFP-positive cells. The data shown are means ± SD from three independent experiments (300 cells were counted per condition). ***P < 0.001 versus cells transfected with GFP alone (Student’s *t* test)

Non-structural protein 3 (NS3) of the Bluetongue virus, a dsRNA virus, has been localized to the Golgi apparatus and shown to specifically modulate the type I IFN signaling pathway [31, 32]. We confirmed that NS3 expression led to the detection of this protein at the Golgi apparatus (Fig. 7a) and that, in luciferase assays, NS3 affected the stimulation of the IFNβ promoter but not NF-κB activation after RLR stimulation (Fig. 7b). Accordingly, NS3 expression decreased the phosphorylation of both TBK1 and IRF3 (Fig. 7c). As NS3 was targeted to the Golgi apparatus and decreased TBK1 activation, we then hypothesized that NS3 binds to OPTN to prevent it from activating TBK1. Immunoprecipitation experiments demonstrated that NS3 binds to OPTN (Fig. 7d) and, in cells expressing NS3, the association between OPTN and TBK1 was impaired after viral infection (Fig. 7e), accounting for the lower levels of TBK1 activation observed (Fig. 7c). Finally, TBK1 aggregation was inhibited in the presence of the viral protein, confirming its ability to neutralize the activity of OPTN (Fig. 7f). Thus, the fact that OPTN is targeted by a viral protein to dampen type I IFN signaling reinforces our findings that OPTN is an important effector in TBK1 activation.

## Discussion

Viral RNAs in endosomes are detected by TLR3, whereas those in the cytosol are detected by RLRs [2]. The stimulation of either of these PRRs leads to TBK1 activation and this kinase plays a crucial role in innate antiviral immunity through the phosphorylation of IRF3, which is required for the production of type I IFNs [7–9]. However, the precise molecular mechanisms underlying TBK1 activation are unclear. Surprisingly, after the stimulation of cells with IL-1β or TNFα, after mitophagy induction or in cancer dependent on KRAS signaling, TBK1 is phosphorylated whereas IRF3 is not [25, 26, 33, 34]. It has been therefore suggested that TBK1 autoactivation and substrate specificity are both dependent on the subcellular distribution of TBK1, with various adaptor proteins each directing TBK1 to discrete signaling complexes for different cellular responses [12, 15, 16]. Consistent with this hypothesis, we observed that the active form of TBK1 is present at the Golgi apparatus after the stimulation of RLRs or TLR3, and that its substrate, IRF3, is phosphorylated. In the case of mitophagy, p-TBK1^S172^ is recruited to depolarized mitochondria without IRF3 phosphorylation [25]. No significant accumulation of active TBK1 was detected at the Golgi apparatus after the treatment of cells with IL-1β or TNFα (data not shown), further suggesting that the presence of p-TBK1^S172^ at the Golgi apparatus is a prerequisite for the phosphorylation of IRF3 by this kinase.

A recent study has elegantly demonstrated that the adaptor proteins MAVS and TRIF (but also STING) harbor conserved domains that are phosphorylated by IKKβ and/or TBK1 in response to stimulation [34]. Phosphorylated adaptor then binds to a positively charged surface of IRF3, thereby recruiting this transcription factor for its phosphorylation and activation by TBK1 [34]. While localized at the Golgi apparatus, active TBK1 does have the possibility to phosphorylate either MAVS or TRIF to promote IRF3 recruitment and ensuing activation of this transcription factor. Indeed, mitochondria (where MAVS anchors) and endosomes (where TRIF is recruited after TLR3 stimulation) through their dynamics or through their trafficking, respectively, are always in close proximity to the Golgi apparatus therefore permitting TBK1 to phosphorylate the adaptor proteins, a process required for IRF3 activation [34]. IFR3 phosphorylation around the Golgi apparatus, an organelle bordering the nucleus where this transcription factor migrates after activation, therefore reduces the possibility of IRF3 to meet phosphatases or E3 ubiquitin ligases that negatively regulate signaling.

A K63-linked polyubiquitination on lysines 30 and 401 is required in the process of TBK1 activation to permit this kinase to phosphorylate IRF3 [13, 14]. Our demonstration that this ubiquitination leads to the targeting of TBK1 to the Golgi apparatus for activation suggests that the ubiquitination sites of TBK1 (this kinase possesses more than 50 lysine residues conserved between humans and mice) might affect the subcellular distribution of this protein and the resulting signaling. It would be interesting to determine whether TBK1 is ubiquitinated after mitochondrial depolarization and, if so, on which lysine residues, for its targeting and activation at the mitochondria for mitophagy, as recently described [25, 26].

One key finding of this study is that ubiquitinated TBK1 is sensed by OPTN at the Golgi apparatus, promoting its *trans*-autophosphorylation after RLR or TLR3 stimulation. The binding of the K63-linked polyubiquitin chains on TBK1 to the UBD of OPTN probably triggers the oligomerization of TBK1-OPTN complexes, resulting in *trans*-autophosphorylation and TBK1 activation, as previously suggested [11] (Additional file 8). OPTN therefore appears to be a positive regulator of TBK1 activation through its polyubiquitin binding activity, as previously described [24, 27]. However, two other studies have suggested that OPTN negatively regulates TBK1 activation after RNA virus infection [35, 36]. We currently have no explanation for this discrepancy. Indeed, OPTN silencing with multiple siRNAs in HEK293T, HeLa cells, or MEFs, and OPTN knockout with CRISPR/Cas9 technology in HeLa cells, MEFs, and BMDMs from OPTN-deficient mice clearly demonstrated an important role of this adaptor in TBK1 activation after RLR and TLR3 stimulation. Consistent with the involvement of OPTN in TBK1 activation, it has recently been reported that OPTN, again through its polyubiquitin binding activity, is required for TBK1 activation in response to mitochondrial depolarization [25, 26]. Moreover, during the preparation of this manuscript, a study reported that osteoclast precursors from OPTN mutant mice produced abnormally low levels of IFNβ in response to RANKL [37]. Additionally, another study reported that BMDM from OPTN^–/–^ mice displayed impaired IRF signaling and low levels of type I IFN production in response to poly(I:C) [28]. Finally, further evidence for the involvement of OPTN in TBK1 activation is provided by our finding that the NS3 protein of the Bluetongue virus targets OPTN at the Golgi apparatus, decreasing TBK1 activation and the resulting IRF3 signaling. Thus, this RNA virus prevents OPTN from activating TBK1 as part of a strategy to modulate the innate immune response to facilitate its replication after infection. Consistent with this finding, other viruses have evolved strategies for targeting the adaptor proteins involved in TBK1 activation. The Vaccinia virus protein C6 binds TBK1 adaptor proteins (TANK, NAP1, and SINTBAD), thereby inhibiting the activation of IRF3 and IRF7 [38], and the Gn protein of hantaviruses disrupts the formation of TBK1 complexes, thereby blocking downstream responses [39].

In addition to acting as a sensor for ubiquitinated TBK1 at the Golgi apparatus for the kinase activation, OPTN has also been reported to be a substrate of TBK1. Indeed, phosphorylation of the Ser177 residue of OPTN by TBK1 increases the association of OPTN with LC3 during xenophagy for the clearance of cytosolic *Salmonella* and the restriction of intracellular bacterial proliferation [40]. Two independent studies have reported that RNA polymerase III detects DNA of viral or bacterial origin in the cytoplasm and induces type I IFNs via the RLR pathway [41, 42]. It therefore seems possible that stimulation of the RLR pathway after bacterial infection promotes TBK1 activation by OPTN at the Golgi apparatus and that, on the one hand, p-TBK1^S172^ triggers the production of type I IFNs and, on the other, the active kinase phosphorylates OPTN to induce the xenophagy of the invading bacteria.

OPTN is associated to Golgi apparatus through an interaction with Rab8 and the interacting domain of OPTN is localized between amino acids 141–209, comprising therefore Ser177 [43]. After TBK1 activation, the phosphorylation of OPTN [40, 44] may disrupt the association between OPTN and Rab8, therefore allowing TBK1-OPTN complexes [44] to leave the Golgi membranes to phosphorylate MAVS or TRIF at the mitochondria or endosomes, respectively, for IRF3 activation [34]. Our observation that p-TBK1^S172^ accumulates at the centrosome at late time points after RLR activation strongly suggests that the active kinase is released from the Golgi membranes after its initial *trans*-autophosphorylation at this organelle.

Other adaptors, such as NAP1 or SINTBAD, have been reported to be involved in TBK1 activation during innate antiviral immunity [45, 46]. We found that, like OPTN, both NAP1 and SINTBAD were partially localized at the Golgi apparatus (Additional file 5C), providing further evidence for a critical role of this organelle in TBK1 signaling during innate immunity. Further studies are required to determine whether and how these adaptors act in concert at the Golgi apparatus to promote TBK1 activation. Indeed, the weak but nevertheless present, IFN-β response in OPTN-deficient cells suggests that factors other than OPTN contribute to TBK1 activity.

## Conclusions

At the Golgi apparatus, we propose that, after RNA viral sensing, OPTN recruits ubiquitinated TBK1 via its UBD, leading to the *trans*-autoactivation of this kinase for the production of type I IFNs after IRF3 phosphorylation (Additional file 8). Our recent report that the Golgi-anchored E3 ubiquitin ligase RNF121 is a new player in the signaling leading to NF-κB activation [47] suggests that the Golgi apparatus, in addition to its other known roles, acts as a hub for the formation and/or maturation of signalosomes, leading to activation of the IRF3 and NF-κB signaling pathways. It remains to be determined whether other signaling pathways are relayed by the Golgi apparatus.

## Methods

### Cell culture and reagents

HEK293T and HeLa cells were obtained from ATCC. MEFs and HEK293T cells stably expressing TLR3 were obtained from Invivogen (San Diego, CA, USA). STING^–/–^, MAVS^–/–^, and TBK1 ^–/–^ MEFs were kindly provided by Dr. Glen Barber (Department of Cell Biology and Sylvester Comprehensive Cancer Center, University of Miami School of Medicine, Miami, USA), Dr. Jurg Tschopp (Department of Biochemistry, University of Lausanne, Epalinges, Switzerland), and Dr. Katherine Fitzgerald (Division of Infectious Diseases, University of Massachusetts Medical School, Worcester, USA), respectively. Primary MEFs isolated from WT or OPTN^470T^ mice were donated by Dr. Ivana Munitic. All cells were cultured in standard conditions. Bone marrow cells from WT or OPTN^470T^ mice were allowed to differentiate in complete DMEM supplemented with 20 % M-CSF-conditioned medium for 6–7 days.

The TLR3 agonist Poly(I:C) was obtained from Invivogen. The Sendai virus H4 strain and the vesicular stomatitis virus mutant strain used to infect cells (multiplicity of infection = 5) and to stimulate the RLRs were provided by Dr. Dominique Garcin. Complexes between low or high molecular weight poly(I:C) and the transfection reagent Lyovec (Invivogen) were also used to activate RLRs.

### Subcellular fractionation

For subcellular organelle fractionation, cells were mechanically disrupted with a 27G1/2 syringe (BD Biosciences, East Rutherford, NJ, USA) in H60 buffer (20 mM HEPES pH 7.9, 1.5 mM MgCl_2_, 60 mM KCl) supplemented with protease inhibitor cocktail (Thermo Scientific, Illkirch, France). Samples were centrifuged at 1000 × *g* to remove the nuclei and unbroken cells. The supernatant (S1) was centrifuged at 5000 × *g* for 5 minutes, to precipitate heavy organelles (P5). The supernatant (S5) was further centrifuged at 10,000 × *g* for 10 minutes to generate S10 and P10. S10 was centrifuged at 25,000 × *g* for 20 minutes to obtain the cytosolic fraction (S25) and P25. Each pellet was resuspended in lysis buffer and analyzed by immunoblotting. Discontinuous Optiprep gradients were used to separate the fractions, as previously described [18]. Isolation of Golgi membranes was performed using a Golgi isolation kit (Sigma Aldrich, St. Louis, MO, USA) according to the manufacturer’s instructions.

### In vitro IRF3 phosphorylation assays

Cells were disrupted in hypotonic buffer (10 mM Tris/HCl pH 7.5, 10 mM KCl, 0.5 mM EGTA, 1.5 mM MgCl_2_, plus protease inhibitor cocktail); 5 to 15 μg of P5 or P25 in resuspension buffer (20 mM HEPES-KOH pH 7.4, 10 % glycerol, 0.5 mM EGTA, and protease inhibitor cocktail) were mixed with 1 μg of recombinant GST-IRF3 (Abnova, Taipei City, Taiwan) and reaction buffer (20 mM HEPES-KOH pH 7.0, 2 mM ATP, 5 mM MgCl_2_ and protease inhibitor cocktail) at 30 °C. The mixture was then cleared by centrifugation and the supernatant was used for immunoblotting.

### Immunofluorescence

Cells were grown on coverslips. They were fixed by incubation in 4 % paraformaldehyde in phosphate buffered saline (PBS) for 10 minutes, and then permeabilized by incubation with 0.15 % Triton X-100 in PBS for 15 minutes. Non-specific binding sites were blocked by incubating cells in a solution of 2 % BSA in PBS for 1 hour. The cells were then incubated for 1 hour at room temperature or overnight at 4 °C with the primary antibodies. They were washed three times, for five minutes each, in PBS and were then incubated for 1 hour with the specific Alexa Fluor-conjugated secondary antibodies (Invitrogen, Life Technologies, Grand Island, NY, USA). Nuclei were stained with DAPI (Sigma) and cells were again washed three times with PBS. Images were acquired with a Leica SP5 confocal microscope (Leica Microsystems, Wetzlar, Germany) equipped with a 63× oil immersion fluorescence objective.

### DNA transfection and plasmids

HEK293T cells were transfected using Fugene 6 (Promega, Madison, WI, USA), according to the manufacturer’s instructions. pcDNA-Myc-TBK1^K38M^ and pcDNA-Myc-TBK1^K30R/K401R^ were obtained by PCR-directed mutagenesis from pcDNAMyc-TBK1. pLEX-TBK1, pLEX-TBK1^K38M^, and pLEX-TBK1^K30R/K401R^ were kindly provided by Dr. Michael Eck (Department of Cancer Biology, Dana-Farber Cancer Institute, Boston, USA). Finally, plasmids encoding NS3-GFP and OPTN-Flag were kindly donated by Dr. Damien Vitour and Dr. Ivana Munitic, respectively.

### Protein extraction, immunoprecipitation and immunoblots

Cells were lysed in lysis buffer (50 mM Tris-HCl pH 7.4, 150 mM NaCl, 1 % Triton X-100, 2 mM EDTA, 2 mM sodium pyrophosphate, 25 mM β-glycerophosphate, 1 mM sodium orthovanadate) supplemented with protease inhibitor cocktail (Thermo Scientific), and the debris were removed by centrifugation at 10,000 × *g* and 4 °C. Protein concentration was determined with a micro BCA kit (Thermo Scientific). For immunoprecipitation, the samples were precleared with protein-G-Sepharose beads (Roche) for 30 minutes before immunoprecipitation with 2.5 μg antibodies and additional protein-G-Sepharose beads at 4 °C for 2 hours. Samples were then boiled in SDS sample buffer (Novex, San Diego, CA, USA) containing 10 % β-mercaptoethanol (Sigma Aldrich) and resolved by SDS-polyacrylamide gel electrophoresis. The cells were lysed and their ubiquitin conjugate content was analyzed at room temperature in denaturing conditions (8 M urea, 0.1 M NaH_2_PO_4_, 10 mM Tris-HCl pH 8, 1 % Triton X-100, 1 % NP-40, 20 mM imidazole). Immunoblot analysis was performed with specific antibodies and the antigen**–**antibody complexes were visualized by chemiluminescence (Immobilon Western, Merck Millipore, Billerica, MA, USA).

### Antibodies

The primary antibodies used for immunoblotting were rabbit monoclonal anti-TBK1 (Abcam, Cambridge, UK, Abcam Cat# ab40676 RRID:AB_776632, 1:5000 dilution), rabbit monoclonal anti-phosphorylated TBK1 (phospho S172) (Abcam Cat# ab109272 RRID:AB_10862438, 1:2000), rabbit monoclonal anti-phosphorylated TBK1 (phospho S172) (Cell Signaling Technology, Danvers, MA, USA, Cell Signaling Technology Cat# 5483P RRID:AB_10693472, 1:2000), rabbit monoclonal anti-IRF3 (Cell Signaling Technology Cat# 4302S RRID:AB_1904036, 1:5000), rabbit monoclonal anti-phosphorylated IRF3 (phospho S386) (Abcam Cat# ab76493 RRID:AB_1523836, 1:5000), rabbit monoclonal anti-phosphorylated IRF3 (phospho S396) (Cell Signaling Technology Cat# 4947S RRID:AB_823547, 1:2000), mouse monoclonal anti-MAVS (Enzo Life Sciences, Farmingdale, NY, USA, Enzo Life Sciences Cat# ALX-804-847 RRID:AB_10539976, 1:2000), rabbit polyclonal anti-TRAF3 (Santa Cruz Biotechnology, Santa Cruz, CA, USA, Santa Cruz Biotechnology Cat# sc-1828 RRID:AB_2209427, 1:1000), rabbit polyclonal anti-HA (Sigma-Aldrich Cat# H6908 RRID:AB_260070, 1:2000), rabbit polyclonal anti-TRIF (Cell Signaling Technology Cat# 4596S RRID:AB_2256555, 1:2000), rabbit polyclonal anti-GAPDH (Sigma-Aldrich Cat# G9545 RRID:AB_796208, 1:20000), rabbit polyclonal anti-KTN (Santa Cruz Biotechnology Cat# sc-33562 RRID:AB_2133047, 1:10000), mouse monoclonal anti-LAMP2 (Santa Cruz Biotechnology Cat# sc-18822 RRID:AB_626858, 1:10000), mouse monoclonal anti-EEA1 (BD Biosciences, East Rutherford, NJ, USA, BD Biosciences Cat# 610456 RRID:AB_397829, 1:10000), rabbit monoclonal anti-Syntaxin6 (Cell Signaling Technology Cat# 2869S RRID:AB_2196500, 1:5000), rabbit polyclonal anti-VDAC (Millipore Cat# AB10527 RRID:AB_10806766, 1:10000), mouse anti-glutathione-S-transferase (BD Biosciences Cat# 554805 RRID:AB_395536, 1:2000), mouse monoclonal anti-Lys-63 specific (Millipore Cat# 05-1313 RRID:AB_1587585, 1:500), mouse monoclonal anti-actin (Sigma-Aldrich Cat# A3853 RRID:AB_262137, 1:10000), rabbit polyclonal anti-optineurin (Abcam Cat# ab23666 RRID:AB_447598, 1:5000), rabbit polyclonal anti-optineurin (Cayman Chemical, Ann Arbor, MI, USA, Cayman Chemical Cat# 100000 RRID:AB_10078198, 1:4000), mouse monoclonal anti-optineurin (Santa Cruz Biotechnology Cat# sc-166576 RRID:AB_2156554, 1:2000), mouse anti-IKKγ (NEMO) (BD Biosciences Cat# 611306 RRID:AB_398832, 1:2000), rabbit polyclonal anti-ERK1/2 (Cell Signaling Technology Cat# 9102 RRID:AB_330744, 1:5000), mouse monoclonal anti-phosphorylated ERK1/2 (Cell Signaling Technology Cat# 9106 RRID:AB_331768, 1:2000), rabbit polyclonal anti-IκBα (Cell Signaling Technology Cat# 9242 L RRID:AB_823540, 1:5000), mouse monoclonal anti-phosphorylated-IκBα (Cell Signaling Technology Cat# 9246 L RRID:AB_226714, 1:2000), mouse monoclonal anti-GFP (Roche Molecular Biochemicals, Basel, Switzerland, Roche Cat# 11814460001 RRID:AB_390913, 1:2000), mouse monoclonal anti-GM130 (BD Biosciences Cat# 610822 RRID:AB_398141, 1:4000), and mouse monoclonal anti-giantin (Abcam Cat# ab37266 RRID:AB_880195, 1:2000).

The primary antibodies used for immunofluorescence were rabbit monoclonal anti-phosphorylated TBK1 (phospho S172) (Abcam Cat# ab109272 RRID:AB_10862438, 1:500 dilution), rabbit monoclonal anti-phosphorylated TBK1 (phospho S172) (Cell Signaling Technology Cat# 5483P RRID:AB_10693472, 1:250), mouse monoclonal anti-IKKε/TBK1 (Cayman Chemical Cat# 13929 RRID:AB_10679144, 1:500), rabbit monoclonal anti-TBK1 (Abcam Cat# ab40676 RRID:AB_776632, 1:500), rabbit monoclonal anti-IRF3 (Cell Signaling Technology Cat# 4302S RRID:AB_1904036, 1:500), rabbit polyclonal anti-IRF3 (Santa Cruz Biotechnology Cat# sc-9082 RRID:AB_2264929, 1:500), mouse monoclonal anti-GM130 (BD Biosciences Cat# 610822 RRID:AB_398141, 1:1000), mouse monoclonal anti-cytochrome c (BD Biosciences Cat# 556432 RRID:AB_396416, 1:1000), mouse monoclonal anti-EEA1 (BD Biosciences Cat# 610456 RRID:AB_397829, 1:500), mouse monoclonal anti-ubiquitin (Millipore Cat# ST1200-100UG RRID:AB_2043482, 1:200), mouse monoclonal anti-giantin (Abcam Cat# ab37266 RRID:AB_880195, 1:1000), rabbit polyclonal anti-optineurin (Abcam Cat# ab23666 RRID:AB_447598, 1:500), rabbit polyclonal anti-optineurin (Cayman Chemical Cat# 100000 RRID:AB_10078198, 1:500), mouse monoclonal anti-optineurin (Santa Cruz Biotechnology Cat# sc-166576 RRID:AB_2156554, 1:500) rabbit polyclonal anti-AZI2 (NAP1) (Abcam Cat# ab65242 RRID:AB_1140792, 1:500), rabbit monoclonal anti-SINTBAD (Cell Signaling Technology Cat# 8605S RRID:AB_10839270, 1:100), rabbit polyclonal anti-giantin (Abcam Cat# ab24586 RRID:AB_448163, 1 :400), mouse monoclonal anti-mouse pericentrin (BD Biosciences Cat# 611815 RRID:AB_399295, 1:400) and a rabbit polyclonal anti-pericentrin (Abcam Cat# ab4448 RRID:AB_304461, 1:400).

The primary antibodies used for immunoprecipitation were mouse monoclonal anti-optineurin (Santa Cruz Biotechnology Cat# sc-166576 RRID:AB_2156554), mouse monoclonal anti-GFP (Roche Cat# 11814460001 RRID:AB_390913), and rabbit monoclonal anti-TBK1 (Abcam Cat# ab40676 RRID:AB_776632).

### Luciferase assay

HEK293T cells were co-transfected with 50 ng of a construct encoding firefly luciferase under the control of NF-κB or the IFN-β promoter, and 10 ng of the *Renilla* luciferase pRL**–**TK plasmid (Promega). The transfections were performed using Fugene 6 (Promega), in accordance with the manufacturer’s instructions. Transfected cells were collected and luciferase activity was assessed in the dual-luciferase reporter assay (Promega), on a Fluorostar Optima machine (BMG Labtech, Ortenberg, Germany). Each experiment was carried out in triplicate, with firefly fluorescence units normalized with respect to *Renilla* luciferase fluorescence units.

### Retroviral transduction of MEFs

Recombinant retroviruses were produced by the transient transfection of HEK293T cells with the different pLEX-TBK1 plasmids and plasmids containing a cDNA encoding retroviral packaging gene. Supernatants were collected 48 h after transfection, filtered through a 0.45-nm filter and concentrated with PEG-IT (Cell Signaling). After infection, cells were selected in puromycin (1 μg/mL) and further passaged in culture.

### Transfection with siRNA

HEK293T cells were transfected by the calcium phosphate precipitation method, whereas HeLa cells and MEFs were transfected with the Lipofectamine RNAiMAX (Invitrogen, Life Technologies), according to the manufacturer’s instructions. siRNAs were used at a final concentration of 20 nM. Control non-specific siRNAs and the specific siRNAs were purchased from Sigma-Aldrich. The siRNAs used were: OPTN 1 (5′ GCUUCAAGAGGCACACACAdTdT 3′), OPTN 2 (GUUUGAGAUGCAAAGCAAAdTdT), mNEMO (GCGAGUUCAACAAGCUGAAdTdT), OPTN A (GAGUCAUGAGAAUGAGAAAdTdT), OPTN B (CCAUGAAAGGGAGAUUUGAdTdT), OPTN C (CCAAAGAAAGAGUUUCAGAdTdT), OPTN D (GAAAGCAUGCUAUCAGAAAdTdT), OPTN E (CCAAGAAUUACUUCGAACAdTdT), and hNEMO (GAGUCGUUGGAGGCUGCCACUAAGdTdT).

### CRISPR/Cas9

OPTN knockout was achieved in HeLa cells with CRISPR/Cas9 technology, by optineurin double nickase plasmid transfection (Santa Cruz) and puromycin selection, according to the manufacturer’s protocol.

### Real-time quantitative PCR

RNA was extracted from cells in RLT lysis buffer and purified with the QIAGEN RNeasy Mini kit, according to the manufacturer’s protocol. RNA quality was checked by measuring the ratio of optical densities at 260 and 280 nm. Total RNA (1 μg) was used for cDNA synthesis with a Revert Aid H minus First Strand DNA Synthesis kit (Fermentas, Villebon-sur-Yvette, France). cDNA was then amplified by PCR with a LightCycler 480 (Roche Diagnostics, Basel, Switzerland), using SYBR Green (Fast Start DNA Master SYBR- Green I; Roche Applied Science, Roche Diagnostics). Specific primers were used for IL-6, IFNB1, or GAPDH (as the housekeeping gene), under the following conditions: 95 °C for 15 minutes, 40 cycles of 94 °C for 15 s, and at 55 °C for 30 s, then 72 °C for 30 s. Specific primers were designed with the Universal Probe Library System (Roche Applied Science). The sequences of the primers used were as follows: hIL-6 (Forward: GAAAGTGGCTATGCAGTTTGAA, reverse: GAGGTAAGCCTACACTTTCCAAGA), hIFNB1 (CGACACTGTTCGTGTTGTCA, GAAGCACAACAGGAGAGCAA), hGAPDH (AGCCACATC GCTCAGACAC, AATACGACCAAATCCGTTGACT), mIL-6 (GCTACCAAACTGGATATAATCAGGA, CCAGGTAGCTATGGTACTCCAGAA), mIFNB1 (CACAGCCCTCTCCATCAACTA, CATTTCCGAATGTTCGTCCT), and mGAPDH (AGCTTGTCATCAACGGGAAG, TTTGAT GTTAGTGGGGTCTCG). Results are expressed as 2^–ΔCp^, where Cp is the cycle threshold number. Dissociation curves were analyzed after each run, for each amplicon, to assess the specificity of quantification with SYBR Green.

### Enzyme-linked immunosorbent assay (ELISA)

The concentrations of IFN-β and IL-6 released into the cell culture medium were determined by ELISA, with the Verikine IFN-β kit (PBL Assay Science, Piscataway, NJ, USA) and the Quantikine IL-6 kit (R&D Systems, Minneapolis, MN, USA) for human or mouse cells, according to the manufacturer’s protocol.

### Statistical analysis

We carried out *t* tests with Tukey’s post hoc analysis to assess the statistical significance of differences (Prism GraphPad Software), and the *P* values obtained are indicated in the figure legends. Differences were considered to be significant if *P* < 0.05. ****P < 0.0001, ***P < 0.001, **0.001 < *P* < 0.01, *0.01 < *P* < 0.05. ns, not significant. The data shown in each histogram are the means ± SD from three independent experiments.

## Additional files

Additional file 1: p-TBK1^S172^ localizes to the Golgi apparatus then migrates to the centrosome after RLR activation. (A) Scheme of the subcellular fractionation procedure for obtaining the various membrane fractions in Figs. 1a, 2a, and Additional file 2A. (B) MEFs were either left unstimulated (control) or infected with Sendai virus (SeV) for 6 h (+ SeV). p-TBK1^S172^ staining was then analyzed by immunofluorescence. The Golgi apparatus was identified by labeling with an antibody raised against giantin. Scale bars, 10 μm. On the right, enlargement of the framed zone in the overlay. (C) Isolated Golgi membranes from uninfected or SeV-infected MEFs were analyzed by immunoblotting with antibodies against the indicated proteins. ° Corresponds to the p-TBK1 detection from a previous immunoblot. (D) MEFs were either left unstimulated (control) or infected with SeV for 6 or 9 h (+ SeV). p-TBK1^S172^ staining was then analyzed by immunofluorescence. The Golgi apparatus was identified by labeling with an antibody raised against GM130. Scale bars, 10 μm. On the right, enlargement of the framed zone in the overlay. (E) MEFs were either left unstimulated (control) or infected with SeV for 9 h (+ SeV). p-TBK1^S172^ staining was then analyzed by immunofluorescence. The centrosomes were stained with an antibody raised against pericentrin. Scale bars, 10 μm. On the right, enlargement of the framed zone in the overlay. (PDF 759 kb) Additional file 2: The active form of TBK1 localizes to the Golgi apparatus after RLR stimulation. (A) HeLa cells were either left unstimulated or infected with Sendai virus (SeV) for 6 or 8 h. Cells were then fractionated as described in Additional file 1A, and samples were analyzed by immunoblotting with antibodies against the indicated proteins. EEA1, kinectin, LAMP2, GAPDH, syntaxin-6, and VDAC served as loading and purity controls for endosomes, the endoplasmic reticulum, lysosomes, the cytosol, the Golgi apparatus, and mitochondria, respectively. (Ub)n, polyubiquitin. * Indicates non-specific bands. (B–E) HeLa cells were either left unstimulated (control) or infected with SeV for 6 h (+ SeV). The indicated proteins were then analyzed by immunofluorescence staining. The Golgi apparatus was labeled with an antibody against GM130, whereas the mitochondria were identified by immunostaining for cytochrome c. Scale bars, 10 μm. On the right, enlargement of the framed zone in the overlay. (F) HeLa cells were either left unstimulated or infected with SeV for 6 or 8 h. Cell lysates (Lys.) were then subjected to immunoprecipitation (IP) with an antibody against TBK1. Samples were then analyzed by immunoblotting with antibodies against the indicated proteins. * Indicates non-specific bands. (PDF 2378 kb) Additional file 3: Transfection of MEFs with poly(I:C) triggers TBK1 accumulation at the centrosome at late time points. (A) MEFs were either left untreated (MOCK) or transfected with LMW poly(I:C) (5 μg/mL) for 4 h (trPoly(I:C)). TBK1 staining was then analyzed by immunofluorescence. The Golgi apparatus was labeled with an antibody against GM130, whereas TBK1 was detected with a rabbit monoclonal antibody. Scale bars, 10 μm. (B–E) MEFs were either left untreated (MOCK) or transfected with HMW poly(I:C) (5 μg/mL) for 4 h (trPoly(I:C)). The indicated proteins were analyzed by immunofluorescence staining with specific antibodies. The Golgi apparatus was labeled with an antibody against GM130, whereas centrosomes were labeled with an antibody against pericentrin. Scale bars, 10 μm. On the right, enlargement of the framed zone in the overlay. (PDF 1624 kb) Additional file 4: The p-TBK1^S172^ in aggregates is ubiquitinated. MEFs were either left untreated (MOCK) or transfected with HMW poly(I:C) (5 μg/mL) for 4 h (trPoly(I:C)). The indicated proteins were analyzed by immunofluorescence staining with specific antibodies. Scale bars, 10 μm. (PDF 338 kb) Additional file 5: OPTN, NAP1 and SINTBAD are present at the Golgi apparatus where OPTN is in complex with active TBK1 after RLR activation. (A, C) The indicated proteins were analyzed by immunofluorescence with specific antibodies in HeLa cells. Anti-OPTN (a), rabbit polyclonal anti-OPTN from Cayman Corp; anti-OPTN (b), rabbit polyclonal anti-OPTN from Abcam; anti-OPTN (c), mouse monoclonal anti-OPTN from Santa Cruz. The Golgi apparatus was stained with a mouse monoclonal anti-GM130 antibody or with a rabbit polyclonal anti-giantin antibody. Scale bars, 10 μm. (B) HEK293T cells were either left unstimulated or infected with Sendai virus (SeV) for 6 and 8 h. Golgi-enriched fractions were then subjected to immunoprecipitation (IP) with an antibody against OPTN. Samples were then analyzed by immunoblotting with antibodies against the indicated proteins. * Indicates non-specific bands. (PDF 1007 kb) Additional file 6: OPTN silencing impairs IRF3 signaling after RLR or TLR3 activation. (A) HEK293T cells were transfected with a control nonspecific siRNA (NS) or with five individual OPTNspecific siRNAs (OPTN A, B, C, D and E) or a NEMO-specific siRNA. The cells were then transfected, 48 h later, with an IFNβ promoter reporter or with a NF-κB reporter and the *Renilla* luciferase gene as an internal control. Then, 24 h after transfection, cells were either left unstimulated (Unstim) or infected with Sendai virus (+SeV) for 7 h. Luciferase assays were performed and the results were normalized against *Renilla* luciferase activity. The data shown are means ± SD from three independent experiments (analysis of variance and comparison with NS siRNA-transfected cells in Student’s *t*-test). RLU, relative luminescence units. ns, not significant. (B) HEK293T cells were transfected with a control nonspecific siRNA (NS) or with two individual OPTN-specific siRNAs (OPTN A and OPTN B) or a NEMO-specific siRNA. Then, 72 h later, cells were either left unstimulated or infected with Sendai virus (SeV) for 6 or 8 h. Cell lysates were analyzed by immunoblotting with antibodies against the indicated proteins. * indicates nonspecific bands. (C) As in (B) but with HeLa cells. (D) HEK293T cells were transfected with a control nonspecific siRNA (NS) or with two individual OPTN-specific siRNAs (OPTN A and OPTN B) or a NEMO-specific siRNA. Cells were either left unstimulated (Unstim) or infected with VSV (+VSV) for 7 h. Luciferase assays were performed and the results were normalized against *Renilla* luciferase activity. (E) As in (B) but HEK293T cells were infected with VSV. (F) HEK293T-TLR3 cells were transfected as in (A). Cells were either left unstimulated (Unstim) or stimulated with poly(I:C) (10 μg/ml) for 7 h. Luciferase assays were then performed. (PDF 703 kb) Additional file 7: OPTN deficiency does not affect TBK1 ubiquitination. WT or OPTN knockout HeLa cells were either left unstimulated or infected with Sendai virus (SeV) for 6 or 8 h. Cell lysates (Lys.) were subjected to immunoprecipitation (IP) with an antibody against TBK1. Samples were then analyzed by immunoblotting with antibodies against the indicated proteins. ° Corresponds to OPTN detection from a previous immunoblot. (PDF 392 kb) Additional file 8: Working model. After infection, viral RNAs are sensed by RIG-I in the cytosol or TLR3 in the endosomes. The corresponding adaptors, MAVS and TRIF, trigger the formation of specific signalosomes leading to the ubiquitination of TBK1. At the Golgi apparatus, OPTN recruits ubiquitinated TBK1 via its UBD, leading to the *trans*-autoactivation of this kinase. Activated TBK1 then phosphorylates IRF3, leading to the production of type I IFNs. (PDF 912 kb)

## Abbreviations

BMDM: Bone marrow derived macrophages
IFN: Interferon
IRF3: Interferon regulatory factor (IRF)-3
ISREs: IFN-stimulated response elements
MEF: Murine embryonic fibroblast
NF-κB: Nuclear factor-kappaB
PBS: Phosphate buffered saline
PRRs: Pattern-recognition receptors
RLRs: RIG-I-like receptors
TLRs: Toll-like receptors
WT: Wildtype
