# Supplementary figures and images for: The Golgi apparatus acts as a platform for TBK1 activation after viral RNA sensing
Source: BMC Biol. 2016 Aug 18;14:69. doi: 10.1186/s12915-016-0292-z (PMC4991008; doi:10.1186/s12915-016-0292-z)

**A**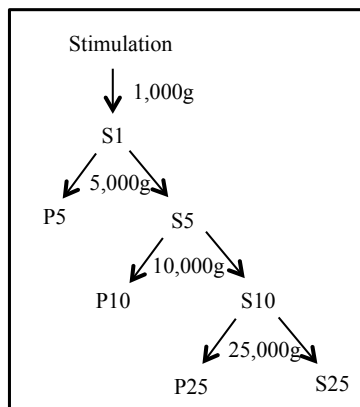**B**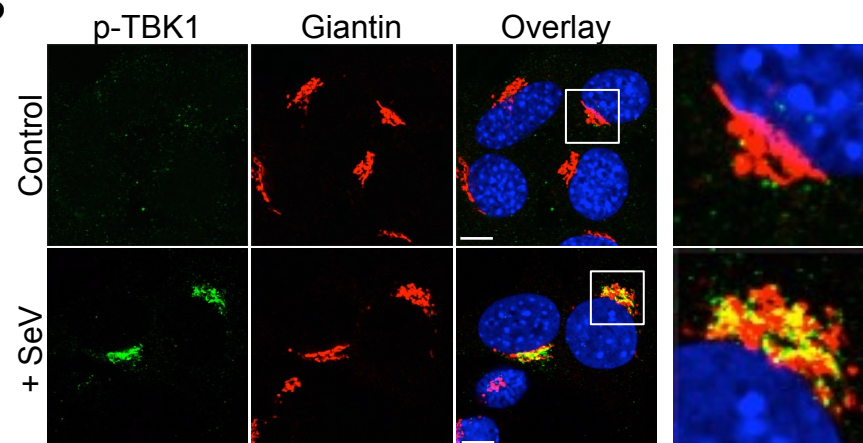**C**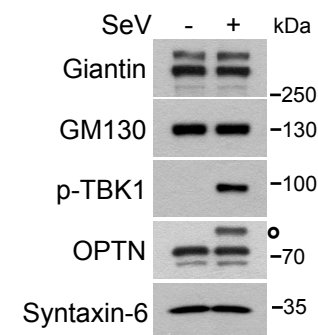**D**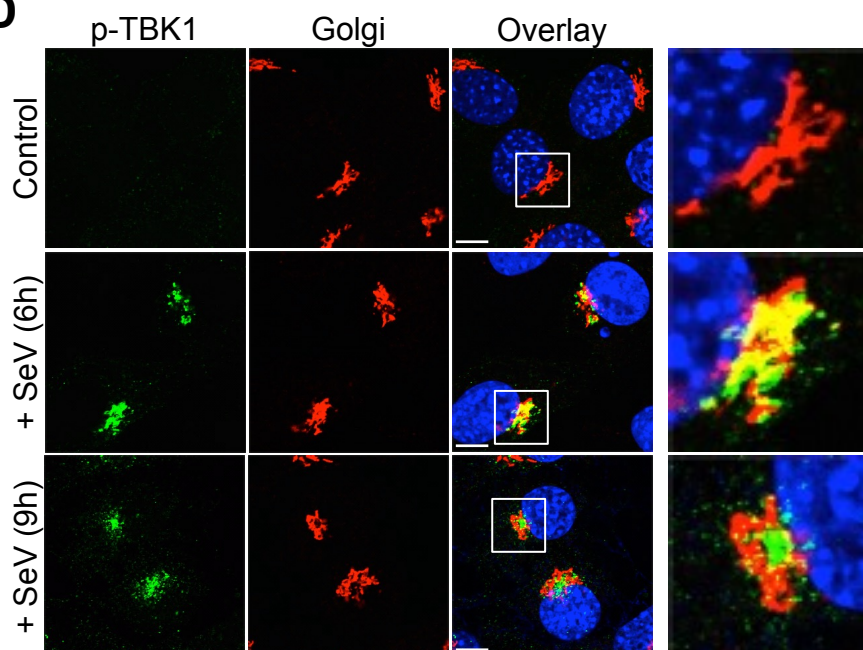**E**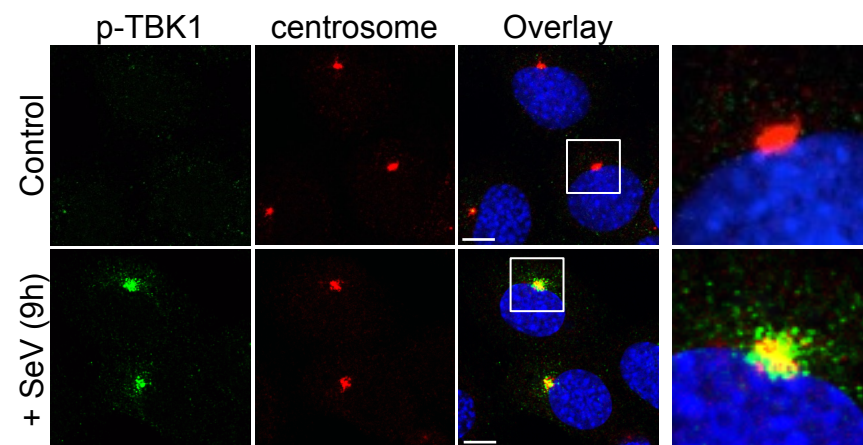

Supplement: Additional file 1: — p-TBK1S172 localizes to the Golgi apparatus then migrates to the centrosome after RLR activation. (A) Scheme of the subcellular fractionation procedure for obtaining the various membrane fractions in Figs. 1a, 2a, and Additional file 2A. (B) MEFs were either left unstimulated (control) or infected with Sendai virus (SeV) for 6 h (+ SeV). p-TBK1S172 staining was then analyzed by immunofluorescence. The Golgi apparatus was identified by labeling with an antibody raised against giantin. Scale bars, 10 μm. On the right, enlargement of the framed zone in the overlay. (C) Isolated Golgi membranes from uninfected or SeV-infected MEFs were analyzed by immunoblotting with antibodies against the indicated proteins. ° Corresponds to the p-TBK1 detection from a previous immunoblot. (D) MEFs were either left unstimulated (control) or infected with SeV for 6 or 9 h (+ SeV). p-TBK1S172 staining was then analyzed by immunofluorescence. The Golgi apparatus was identified by labeling with an antibody raised against GM130. Scale bars, 10 μm. On the right, enlargement of the framed zone in the overlay. (E) MEFs were either left unstimulated (control) or infected with SeV for 9 h (+ SeV). p-TBK1S172 staining was then analyzed by immunofluorescence. The centrosomes were stained with an antibody raised against pericentrin. Scale bars, 10 μm. On the right, enlargement of the framed zone in the overlay. (PDF 759 kb) [file 12915_2016_292_MOESM1_ESM.pdf]

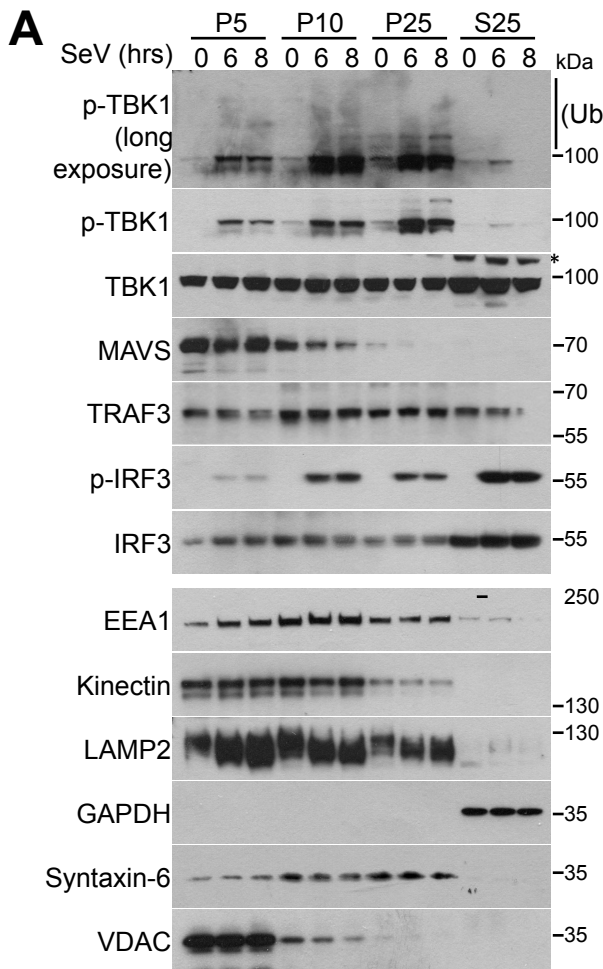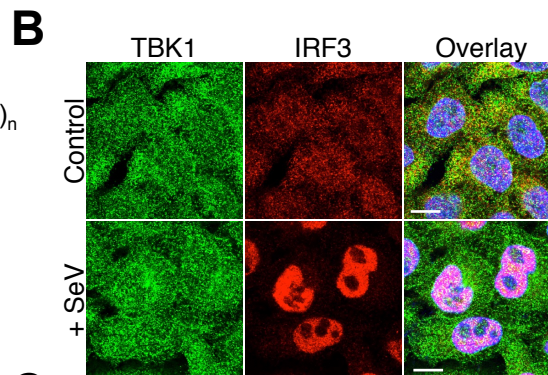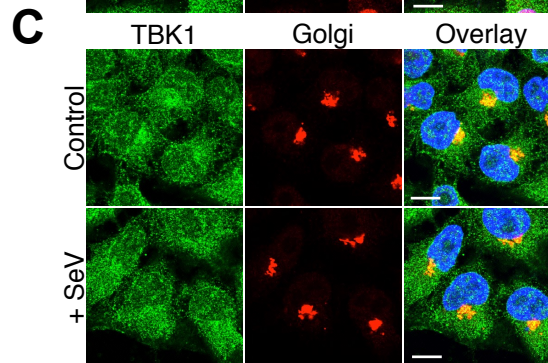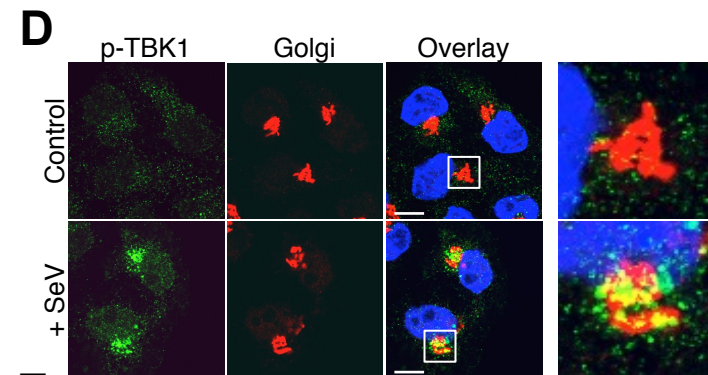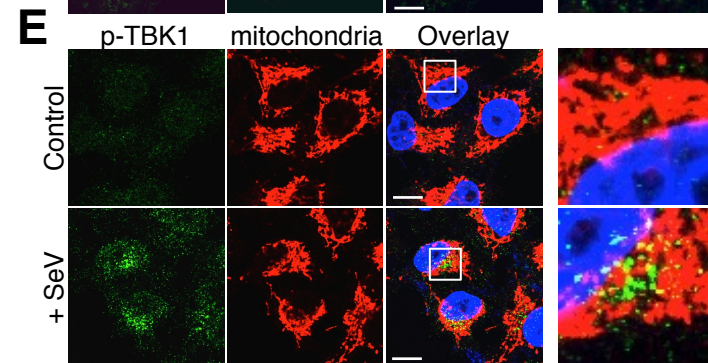

**F**

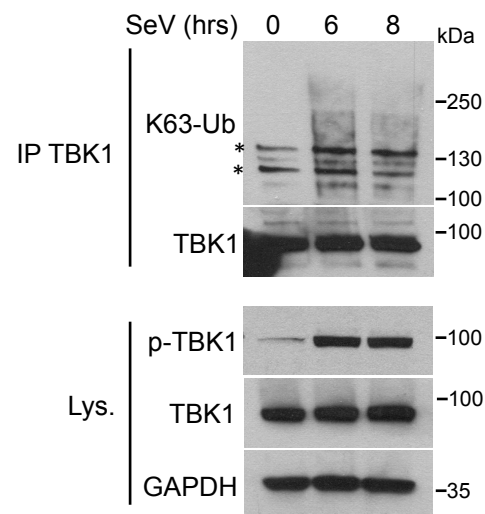

Supplement: Additional file 2: — The active form of TBK1 localizes to the Golgi apparatus after RLR stimulation. (A) HeLa cells were either left unstimulated or infected with Sendai virus (SeV) for 6 or 8 h. Cells were then fractionated as described in Additional file 1A, and samples were analyzed by immunoblotting with antibodies against the indicated proteins. EEA1, kinectin, LAMP2, GAPDH, syntaxin-6, and VDAC served as loading and purity controls for endosomes, the endoplasmic reticulum, lysosomes, the cytosol, the Golgi apparatus, and mitochondria, respectively. (Ub)n, polyubiquitin. * Indicates non-specific bands. (B–E) HeLa cells were either left unstimulated (control) or infected with SeV for 6 h (+ SeV). The indicated proteins were then analyzed by immunofluorescence staining. The Golgi apparatus was labeled with an antibody against GM130, whereas the mitochondria were identified by immunostaining for cytochrome c. Scale bars, 10 μm. On the right, enlargement of the framed zone in the overlay. (F) HeLa cells were either left unstimulated or infected with SeV for 6 or 8 h. Cell lysates (Lys.) were then subjected to immunoprecipitation (IP) with an antibody against TBK1. Samples were then analyzed by immunoblotting with antibodies against the indicated proteins. * Indicates non-specific bands. (PDF 2378 kb) [file 12915_2016_292_MOESM2_ESM.pdf]

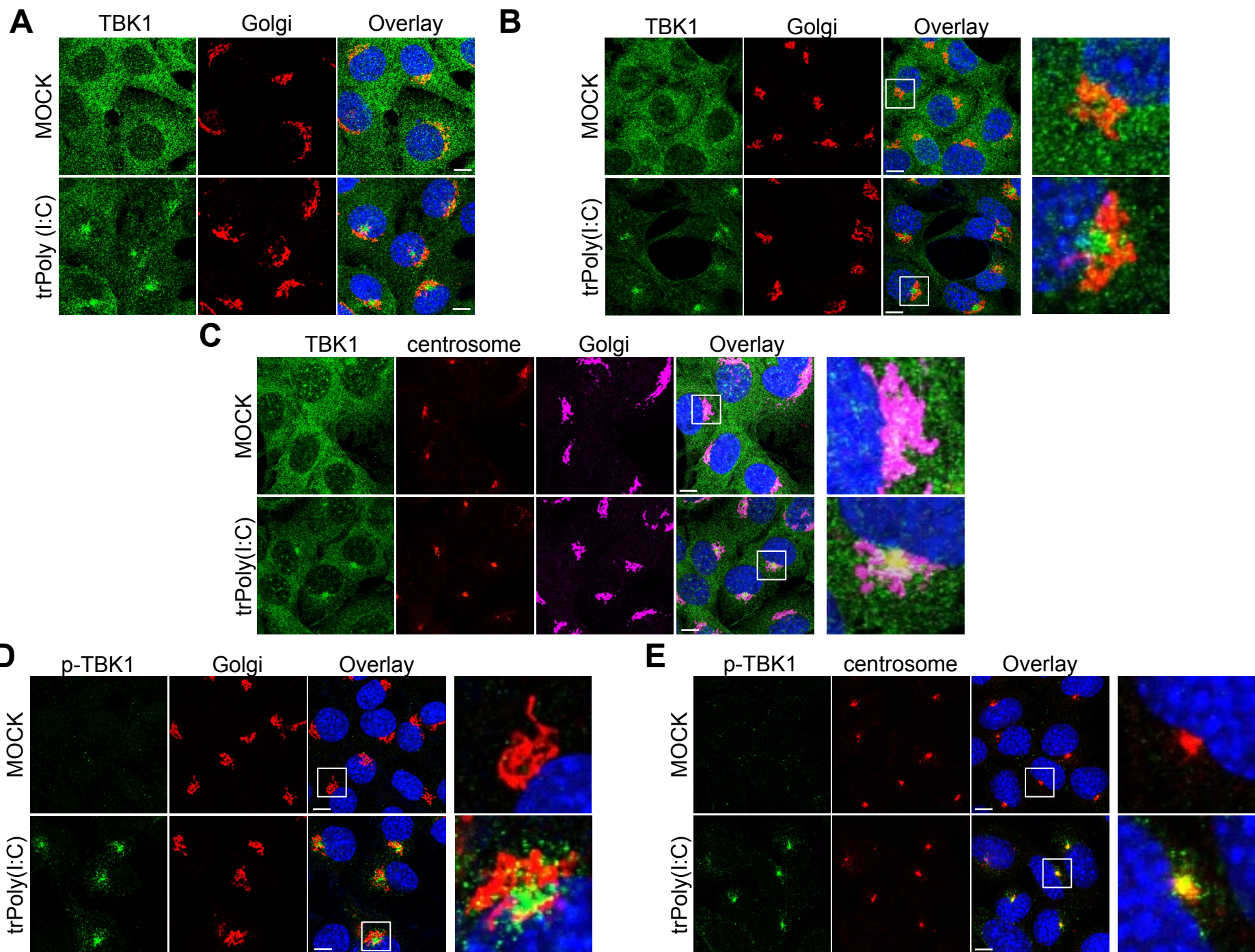

Supplement: Additional file 3: — Transfection of MEFs with poly(I:C) triggers TBK1 accumulation at the centrosome at late time points. (A) MEFs were either left untreated (MOCK) or transfected with LMW poly(I:C) (5 μg/mL) for 4 h (trPoly(I:C)). TBK1 staining was then analyzed by immunofluorescence. The Golgi apparatus was labeled with an antibody against GM130, whereas TBK1 was detected with a rabbit monoclonal antibody. Scale bars, 10 μm. (B–E) MEFs were either left untreated (MOCK) or transfected with HMW poly(I:C) (5 μg/mL) for 4 h (trPoly(I:C)). The indicated proteins were analyzed by immunofluorescence staining with specific antibodies. The Golgi apparatus was labeled with an antibody against GM130, whereas centrosomes were labeled with an antibody against pericentrin. Scale bars, 10 μm. On the right, enlargement of the framed zone in the overlay. (PDF 1624 kb) [file 12915_2016_292_MOESM3_ESM.pdf]

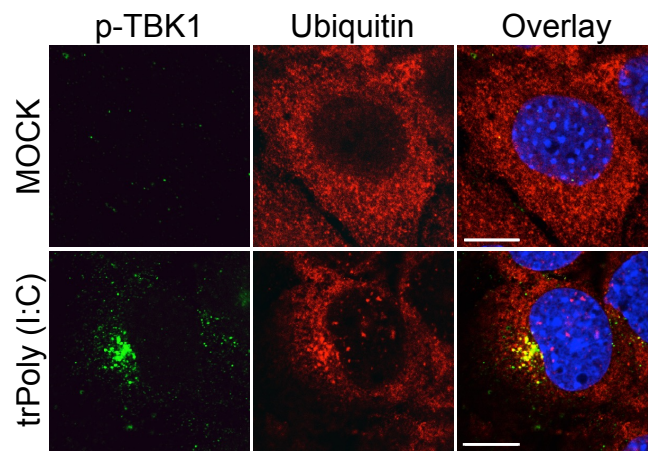

Additional file 4

Supplement: Additional file 4: — The p-TBK1S172 in aggregates is ubiquitinated. MEFs were either left untreated (MOCK) or transfected with HMW poly(I:C) (5 μg/mL) for 4 h (trPoly(I:C)). The indicated proteins were analyzed by immunofluorescence staining with specific antibodies. Scale bars, 10 μm. (PDF 338 kb) [file 12915_2016_292_MOESM4_ESM.pdf]

**A**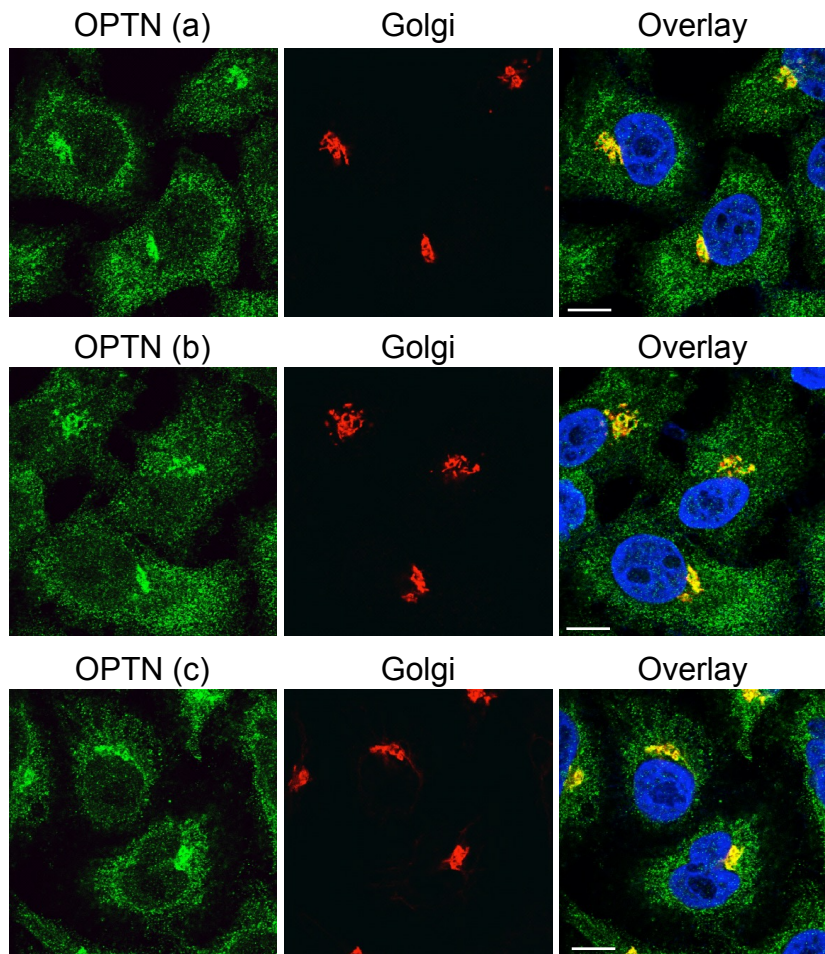**C**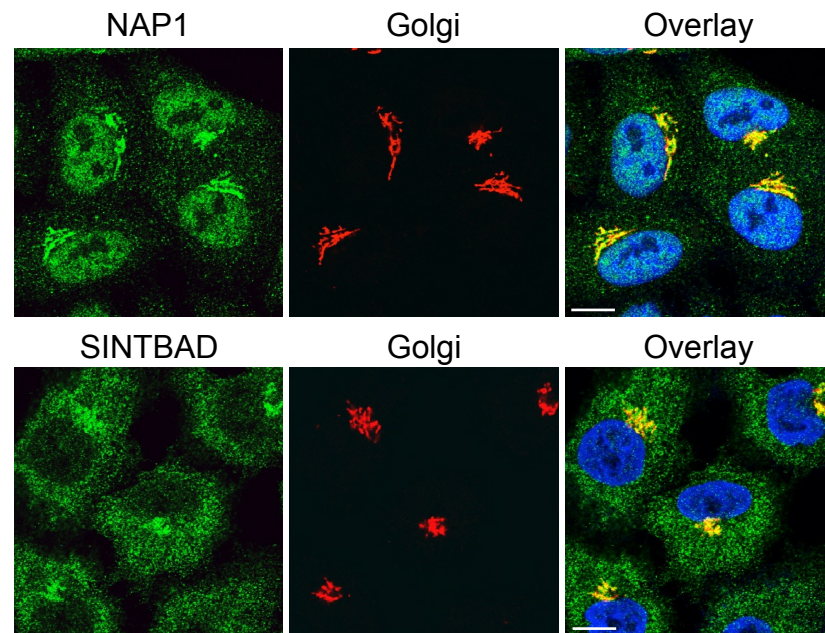**B**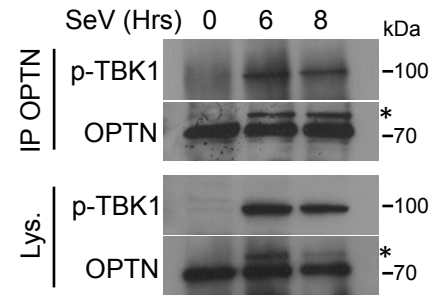

Supplement: Additional file 5: — OPTN, NAP1 and SINTBAD are present at the Golgi apparatus where OPTN is in complex with active TBK1 after RLR activation. (A, C) The indicated proteins were analyzed by immunofluorescence with specific antibodies in HeLa cells. Anti-OPTN (a), rabbit polyclonal anti-OPTN from Cayman Corp; anti-OPTN (b), rabbit polyclonal anti-OPTN from Abcam; anti-OPTN (c), mouse monoclonal anti-OPTN from Santa Cruz. The Golgi apparatus was stained with a mouse monoclonal anti-GM130 antibody or with a rabbit polyclonal anti-giantin antibody. Scale bars, 10 μm. (B) HEK293T cells were either left unstimulated or infected with Sendai virus (SeV) for 6 and 8 h. Golgi-enriched fractions were then subjected to immunoprecipitation (IP) with an antibody against OPTN. Samples were then analyzed by immunoblotting with antibodies against the indicated proteins. * Indicates non-specific bands. (PDF 1007 kb) [file 12915_2016_292_MOESM5_ESM.pdf]

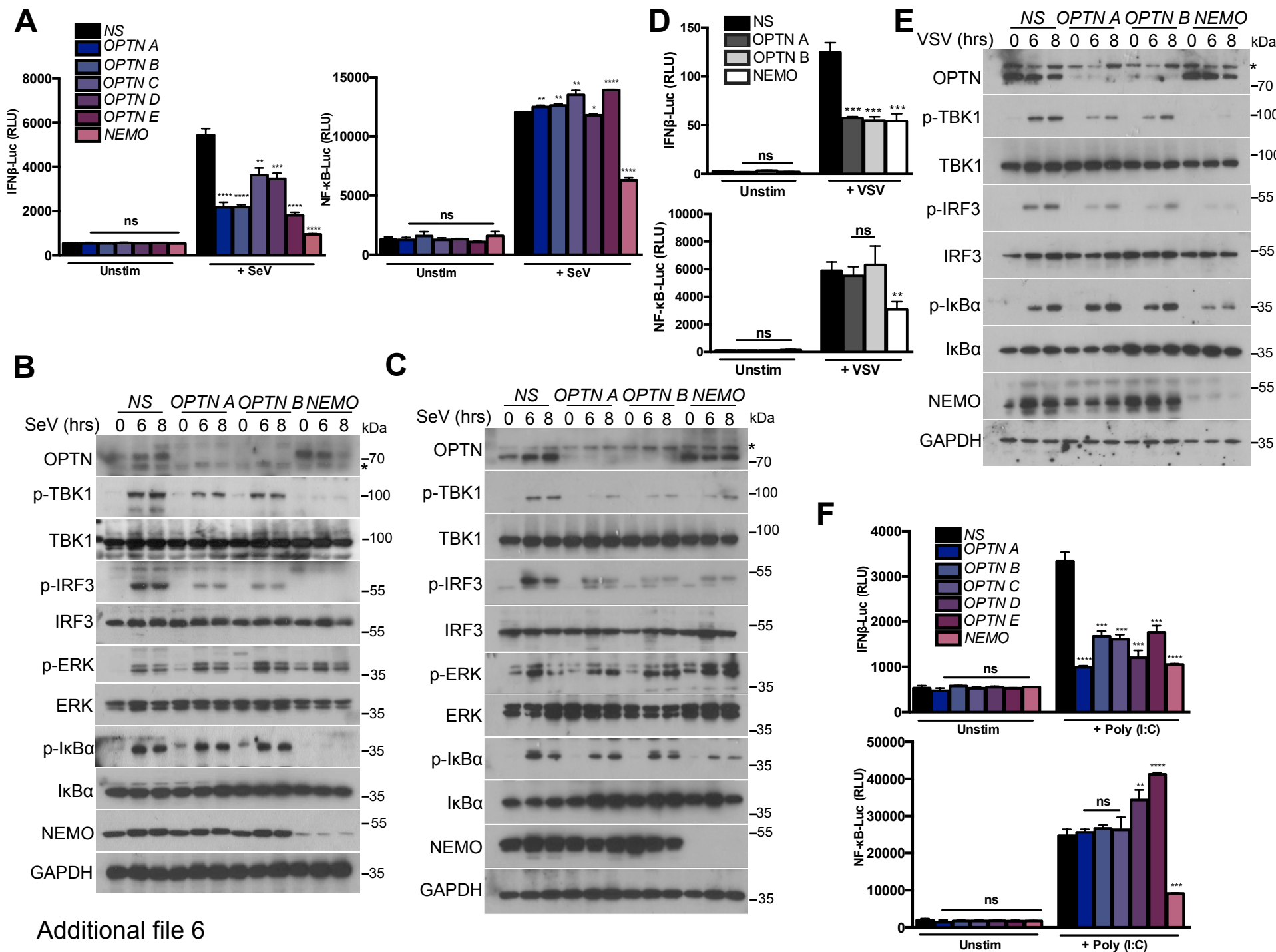

Supplement: Additional file 6: — OPTN silencing impairs IRF3 signaling after RLR or TLR3 activation. (A) HEK293T cells were transfected with a control nonspecific siRNA (NS) or with five individual OPTNspecific siRNAs (OPTN A, B, C, D and E) or a NEMO-specific siRNA. The cells were then transfected, 48 h later, with an IFNβ promoter reporter or with a NF-κB reporter and the Renilla luciferase gene as an internal control. Then, 24 h after transfection, cells were either left unstimulated (Unstim) or infected with Sendai virus (+SeV) for 7 h. Luciferase assays were performed and the results were normalized against Renilla luciferase activity. The data shown are means ± SD from three independent experiments (analysis of variance and comparison with NS siRNA-transfected cells in Student’s t-test). RLU, relative luminescence units. ns, not significant. (B) HEK293T cells were transfected with a control nonspecific siRNA (NS) or with two individual OPTN-specific siRNAs (OPTN A and OPTN B) or a NEMO-specific siRNA. Then, 72 h later, cells were either left unstimulated or infected with Sendai virus (SeV) for 6 or 8 h. Cell lysates were analyzed by immunoblotting with antibodies against the indicated proteins. * indicates nonspecific bands. (C) As in (B) but with HeLa cells. (D) HEK293T cells were transfected with a control nonspecific siRNA (NS) or with two individual OPTN-specific siRNAs (OPTN A and OPTN B) or a NEMO-specific siRNA. Cells were either left unstimulated (Unstim) or infected with VSV (+VSV) for 7 h. Luciferase assays were performed and the results were normalized against Renilla luciferase activity. (E) As in (B) but HEK293T cells were infected with VSV. (F) HEK293T-TLR3 cells were transfected as in (A). Cells were either left unstimulated (Unstim) or stimulated with poly(I:C) (10 μg/ml) for 7 h. Luciferase assays were then performed. (PDF 703 kb) [file 12915_2016_292_MOESM6_ESM.pdf]

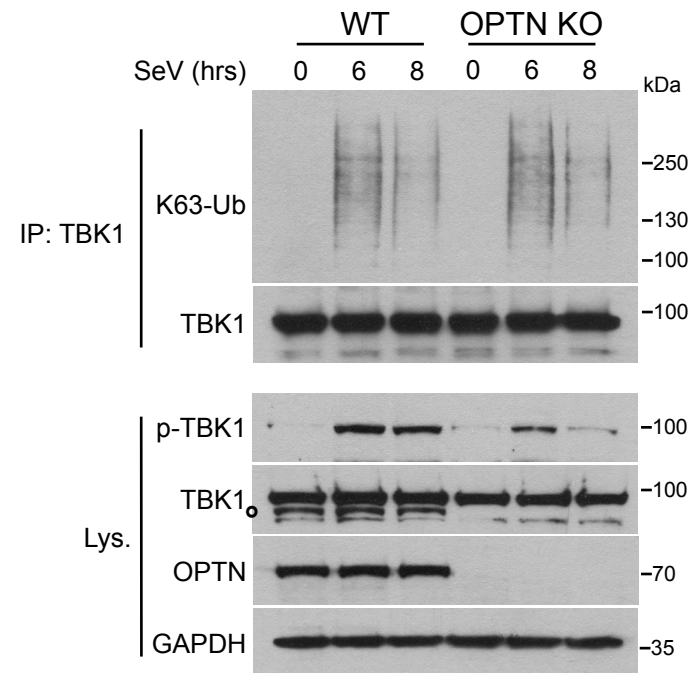

Additional file 7

Supplement: Additional file 7: — OPTN deficiency does not affect TBK1 ubiquitination. WT or OPTN knockout HeLa cells were either left unstimulated or infected with Sendai virus (SeV) for 6 or 8 h. Cell lysates (Lys.) were subjected to immunoprecipitation (IP) with an antibody against TBK1. Samples were then analyzed by immunoblotting with antibodies against the indicated proteins. ° Corresponds to OPTN detection from a previous immunoblot. (PDF 392 kb) [file 12915_2016_292_MOESM7_ESM.pdf]

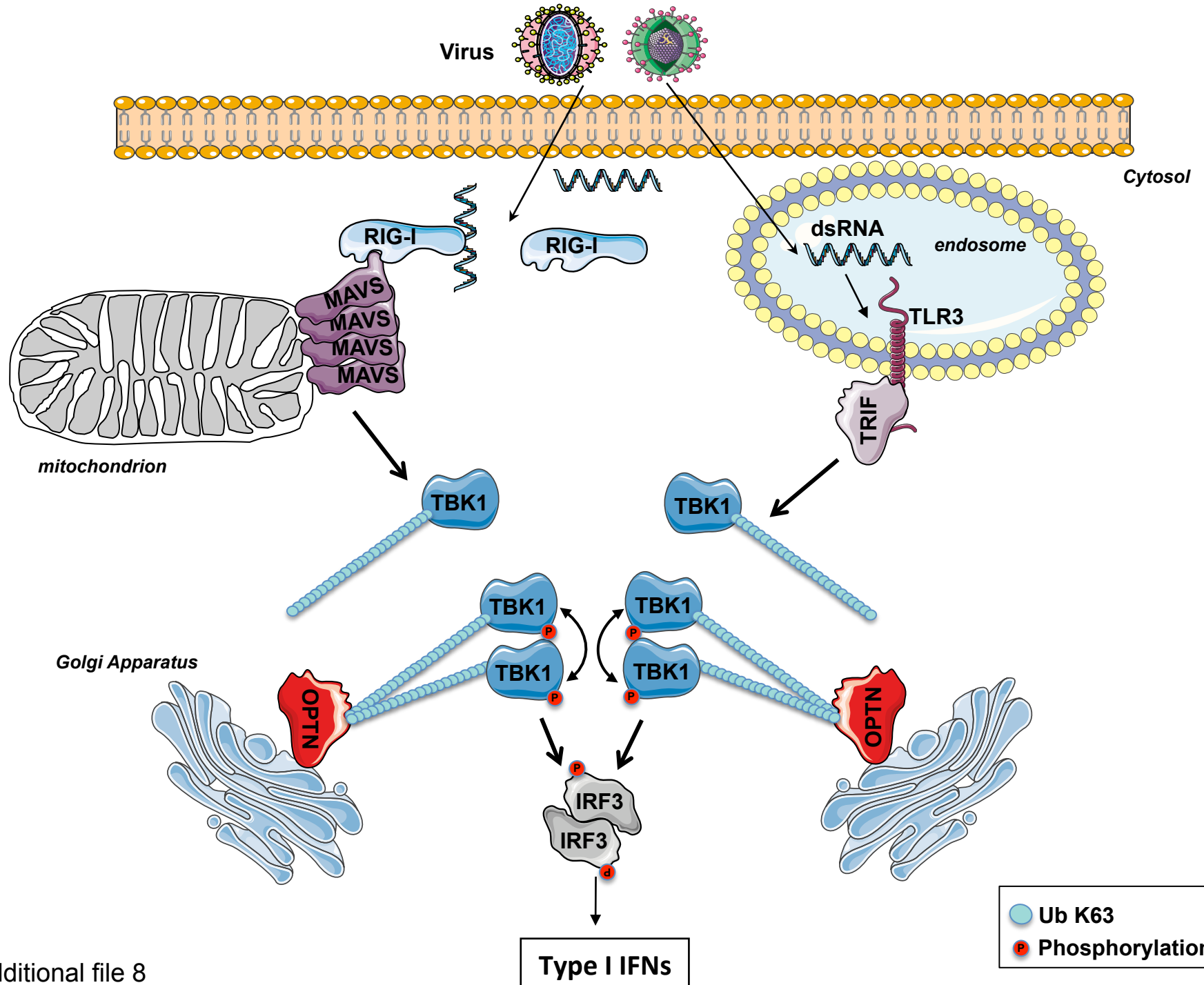

Supplement: Additional file 8: — Working model. After infection, viral RNAs are sensed by RIG-I in the cytosol or TLR3 in the endosomes. The corresponding adaptors, MAVS and TRIF, trigger the formation of specific signalosomes leading to the ubiquitination of TBK1. At the Golgi apparatus, OPTN recruits ubiquitinated TBK1 via its UBD, leading to the trans-autoactivation of this kinase. Activated TBK1 then phosphorylates IRF3, leading to the production of type I IFNs. (PDF 912 kb) [file 12915_2016_292_MOESM8_ESM.pdf]
